# Reassigning sources of misophonic trigger sounds to change their unpleasantness: Testing alternative mechanisms with a new set of movies, paintings, and words

**DOI:** 10.1371/journal.pone.0321594

**Published:** 2025-04-18

**Authors:** Laurie M. Heller, Urszula Oszczapinska, Jessica M. Smith, Megan M. Julien

**Affiliations:** Department of Psychology, Carnegie Mellon University, Pittsburgh, Pennsylvania, United States of America; Universidad Nacional de Tres de Febrero, ARGENTINA

## Abstract

We conducted nine experiments to determine why a sound’s pleasantness can be altered by movies, abstract paintings, and words. In Expt. 1, unpleasant sounds, such as the sound of a person sniffing, were paired either with their original video track or with video tracks depicting neutral events that could plausibly have produced the sound, such as pulling tissues out of a tissue box. While the unpleasant sounds were mildly unpleasant to an unscreened population, these sounds were expected to be more unpleasant for people who have misophonia, a condition in which certain everyday sounds are unbearable. Consistent with past literature, neutral video tracks increased the sounds’ pleasantness for the non-misophonic and misophonic populations, by 0.98 and 1.59 points, respectively (on an 11-point scale). Movies rated as having better audio-visual matches produced greater changes in pleasantness, consistent with the hypothesis that source reassignment caused the changes. Expt. 2 found a consistent result when the video tracks were replaced with written event descriptions, although the effect size was reduced. Expt. 3 inverted Expt. 1 and found that unpleasant video tracks decreased the pleasantness of neutral sounds by 2.12 points, but better-matching movies did not produce greater changes in pleasantness. In Expts. 4–6, we sought an alternative to the source reassignment explanation by obtaining ratings of audio-visual synchrony, cross-modal agreement in symbolism, source plausibility, and sound identifiability. No complete explanation was found for the effect of unpleasant videos. Furthermore, pleasant abstract paintings increased the pleasantness of unpleasant sounds by 0.37 points, correlating with cross-modal agreement but not with audio-visual match. Taken together, different types and patterns of match ratings can help discern the causal mechanisms by which visual stimuli affect sound pleasantness (e.g., source reassignment, cross-modal agreement).

## Introduction

Despite their ubiquity, everyday sounds can elicit a wide range of emotional and physiological responses. Some sounds, such as a babbling brook, will typically evoke feelings of calmness, while other sounds, such as crying, will typically evoke feelings of sadness or discomfort. Although there is general agreement about which environmental sounds are pleasant or unpleasant to most people, there are also profound individual differences that depend upon prior experiences and context. In fact, some everyday sounds that are considered relatively neutral to most people can be unbearable to others. Misophonia [1,2] is a disorder characterized by strong emotional reactions, such as feelings of irritation, rage, and/or disgust, in response to certain everyday sounds, such as chewing, sniffing, or pen clicking. Although common misophonic triggers are produced by oral and nasal regions of the human body, repetitive sounds are also a class of triggers [3]. Despite these common trends, every individual with misophonia has their own unique set of triggers. Reactions can also include physiological responses (e.g., increase in heart rate and perspiration). In severe cases, individuals avoid places where unbearable sounds are likely to be encountered, significantly affecting their overall quality of life [4]. Additionally, a similar emotional reaction can sometimes be triggered by visual images depicting (or labeling) events that would normally produce the trigger sounds [4,5]. Unfortunately, sounds and images that trigger misophonic reactions (hereafter, triggers) are difficult to avoid as they are often encountered in everyday life.

The emotional reaction evoked by a sound depends on many factors, such as the presumed source of the sound (e.g., a specific person), the presumed action producing the sound (e.g., eating), what other sounds are present, and whether the action is socially appropriate (e.g., eating in the library). Although perceptual properties of sounds can influence their pleasantness, they alone are insufficient to determine its emotional impact. For example, a single sound can be heard as either unpleasant or neutral depending on whether its source is correctly identified [6]. For misophonia in particular, reactions to triggers can be reduced by acoustic manipulations that reduce their identifiability, such as adding noise or distortion [7,8]. This motivates the question of whether intentionally changing the identification of a trigger’s source could reduce its negative impact.

There is evidence that the unpleasantness of a trigger can be reduced by the suggestion of an alternative neutral source for the sound. This suggestion has been accomplished in a number of ways: (1) specifying a non-human source of an eating action [9], (2) accompanying a sound with an image or video that implies a different source [10–12], and (3) modifying the interpretation of the source of the sound via text descriptions [11]. However, prior studies have not clarified how to generalize this approach for new sounds by outlining the requirements for an effective alternative neutral source. For example, must the unpleasant sounds be inherently ambiguous? Does the alternative source need to be believable, or even meaningful? We set out to explore the factors that make for effective alternative neutral sources by testing alternative hypotheses about the mechanisms for source reassignment.

When a sound is accompanied by a video depicting its source event, this introduces dynamic temporal factors that can introduce audio-visual incongruence [13]. Therefore, videos designed to produce source reassignment may be more effective if they temporally align with the sound. It is also possible, although speculative, that source reassignment is aided when the visual source matches the intuitive physics of sound production (e.g., a forceful motion should accompany a loud sound [14].) Furthermore, perceptual input that is pleasant but unrelated to the sound source may still make unpleasant sounds more tolerable, such as pleasant music played during meals [15]. Therefore, it is necessary to account for both the related and unrelated accompanying input that affects the context of triggers.

Samermit, Saal, and Davidenko [16] paired brief unpleasant sounds with videos depicting positive alternative sources (PAVS). They compared the pleasantness of the sound alone to a sound accompanied by a PAVS. They found that this sound-video pairing reduced the unpleasantness of the sounds for a general population. They postulated that the PAVS convinced observers that the sounds were produced by the pleasant source and therefore the sounds were perceived as more neutral. However, there are a few threats to the validity of this claim. First, the experimental design did not include a control condition of rating the sounds twice in a row without watching the PAVS, so it is not clear if the unpleasant sounds could have been rated more positively on their second appearance due to the “mere exposure” effect, in which neutral stimuli become more pleasing with repetition [17]. Second, it is possible that the presence of a video distracted attention from sounds, thereby making them less unpleasant [18]. Third, it is possible that the videos were generally pleasing to view and this may have contaminated the participants’ ratings of the sounds [19,20].

Follow-on studies addressed some of these threats to the hypothesis that PAVS cause the source of the sound to be reassigned [11,12]. These studies compared the pleasantness rating of unpleasant sounds when paired either with PAVS or with their original video source. They introduced a new measure that asked how well the video and audio components of the movies appeared to match. Presumably, high match ratings indicate that the audio and video events are plausible and/or synchronous. The pleasantness of sounds paired with PAVS was rated higher when the match was rated higher. This relationship was interpreted as evidence that the better-matching movies (video + audio) were changing the source assignment of the sounds, thereby increasing the sounds’ pleasantness (which we name the *source reassignment hypothesis*). Alternatively, it is possible that the better-matching movies were more pleasant to watch because congruent stimuli are typically more pleasant (cf. [21]), leading to an increase in the sound pleasantness ratings. Furthermore, the sounds that are relatively more pleasant could be more amenable to matching with the pleasant video components, which could explain why the largest benefit was seen for the most pleasant sounds. Therefore, a positive relationship between PAVS sound pleasantness and match quality does not prove that better-matching PAVS caused a greater change in the interpretations of the sounds’ sources. Thus, it is necessary to provide further evidence to estimate how much of the effect of accompanying stimuli (whether videos, words, or images) is due to an alteration in the perceived source of the sound.

Our goal is to understand the beneficial causal mechanisms of viewing alternative sources while listening to unpleasant sounds. Isolating these mechanisms could assist with developing a broader set of stimuli that could potentially be applied to cognitive reframing of unbearable sounds (e.g., in the context of psychotherapy, real-time interventions, or mobile applications [22]). As a first step in accomplishing our goal, we replicated and extended prior studies [16,23] by creating a new set of alternative visual sources for an expanded set of triggers. We compared the pleasantness rating of triggers when paired either with an alternative neutral source or with their original video source. To test predictions of the *source reassignment hypothesis*, we also asked how well the video and audio components of the movies appeared to match, and clarified whether the match was about plausibility, synchrony, or cross-modal sound symbolism. We compared misophonic participants to a non-misophonic control group. We then asked whether the match rating given by both groups correlated with the pleasantness of sounds and/or videos. To address whether movies with *better-matching* neutral sources caused a *greater change* in the interpretations of the sounds’ sources, we asked whether match predicted the *change* in sound pleasantness ratings between the two video conditions.

Our second step was to ask whether *source reassignment* could be accomplished semantically without the use of images. We used simple phrases describing neutral or unpleasant sources for the unpleasant sounds to influence their pleasantness ratings. We compared the size of this semantic effect on source reassignment to the effect from our first study that used accompanying visual input. We quantitatively evaluate how much of the beneficial effect of neutral visual sources could be accomplished by text descriptions of those same sources. Our study was conducted on both a misophonic and non-misophonic group to investigate the possibility that concurrent text descriptions could be a cost-effective alternative for source reassignment when movies are not available.

We created a third way to test the *source reassignment hypothesis* by pairing neutral sounds with unpleasant visual sources. The visual sources, which were videos depicting sources of misophonic triggers, were predicted to cause the sounds to be rated as more unpleasant. These stimuli were useful for disentangling the alternative explanations for the association between pleasantness and match. If a better-matching movie makes the visual source more convincing, then a movie with a better match should have a larger negative effect on sound pleasantness. In contrast, if better-matching movies are more pleasant to watch, then a better audio-video match should increase the sound pleasantness ratings. We also investigated the meaning of a good match rating by evaluating the distinctions between matches based on event plausibility, temporal synchrony, and/or cross-modal agreement in sound symbolism. Because this and subsequent questions addressed a general cognitive mechanism, we tested an unscreened population (i.e., participants were neither included nor excluded based on misophonic status).

A fourth way to test the *source reassignment hypothesis* is to measure the effect of visual pleasantness which is not meaningfully related to the sounds. Because unrelated videos would have mismatched timing in audio and video, static images are the best choice for an unrelated stimulus. We asked whether simply looking at a pleasant image while listening to the misophonic triggers will cause the ratings of the sounds to be more positive than when the sound is heard alone. If so, that effect requires an explanation other than source reassignment. In two parallel experiments, we established how to discriminate between visual pleasantness and source reassignment via patterns of match ratings. Furthermore, we quantitatively compared the effect sizes of the pleasant images and source-reassigning movies.

## General methods

This General methods section begins with an explanation of our movie construction method for all the movies in all the experiments reported herein. This section includes information about recording techniques and devices, editing software, as well as video and audio normalization procedures. It also includes definitions of our participant populations and our data quality procedures.

### Movie construction methods

#### Generating ideas for alternative sources.

To generate a new set of movies visually showing alternative neutral sound sources for misophonic trigger sounds, we first conducted an extensive search of the misophonia literature to compile a list of common triggers. For a sound to be considered a trigger, the sound must be supported by empirical evidence or be self-reported by patients in a published hearing experiment or questionnaire. Based on our search criterion, we found 56 unique classes of misophonic triggers (see S1 Table). The classes of trigger sounds that appeared most frequently in the literature were: general chewing sounds, human vocalizations, and repetitive sounds.

Next, we created a list of alternative neutral sound sources for the unpleasant sounds. In our brainstorming sessions, we varied both the physical interaction and the material properties of the objects that produced the sounds. We created various stimuli to test out ideas, some of which were informed by misidentifications of similar sounds in previous studies in our lab. For all neutral alternatives, we used a source object and action that differed from the unpleasant sound. After in-house pilot testing of plausibility of the alternative sources, we selected the following 20 sounds from the top classes of trigger sounds: *person blowing their nose, person eating chips, person chewing gum, person scratching scalp, person swishing water in their mouth, person crinkling a plastic bottle, person cracking their knuckles, person gulping water, person sucking in air through their teeth, person coughing, person wheezing, person typing on a keyboard, person sneezing, person brushing their teeth, person smacking their lips, person breathing loudly through the nose*, *person sniffing 1,* and *person sniffing 2*. In addition, we included two sounds that are typically considered unpleasant for much of the population: *person scratching a blackboard,* and *person scraping a fork and knife together*. To encompass the variety of these 20 sounds, we refer to them as *unpleasant* sounds rather than trigger sounds.

#### Audio and video recordings.

In the lab, sounds were recorded with a Zoom H4N Pro microphone at a 24-bit/96kHz sampling rate in a double-walled sound attenuating chamber treated with sound-absorbing foam on the walls and ceiling. In the same chamber, the visual source of the event was recorded using a Zoom Q8 video recorder attached to a tripod (unless otherwise noted below). Using movie editing software (Lightworks [24]), each digital movie was separated into two tracks: (1) a silent video track depicting an Unpleasant (*U*_*v*_) or a Neutral (*N*_*v*_) visual source, and (2) an audio track containing an Unpleasant (*U*_*s*_) or Neutral (*N*_*s*_) sound.

After making original recordings of unpleasant sound events, we created movies of their alternative neutral counterparts. The actor making a neutral sound event was simultaneously watching the original movie of the unpleasant sound it was intended to emulate (a technique used by Foley artists [25]). This technique allowed the actor to follow the temporal pattern of the original unpleasant sound to ensure temporal alignment of the sound and visual source. The headphones and/or video screens were not visible in the framing of these movies.

Several of our videos were not recorded in the lab. Some needed to be recorded outdoors. When the soundtrack was poor quality or missing, we replaced it with an in-lab Foley recording or a recording from freesound.org [26] (e.g., *ducks splashing*, *deer eating leaves*, *campfire burning*, *lawn sprinkler spraying water).* The movie of *birds chirping* was downloaded from YouTube.com with no copyright infringement.

All audio track files were wav-format and equalized to have an equal root-mean-squared level using AudioToolbox functions in Matlab [27,28]. The sounds were between 5 and 20 seconds in duration (see File S2). Likewise, all the visual sources were brightness-equalized using FFmpeg [29], and had the same duration as the sounds with which they were paired.

#### Combining the audio and video tracks into a movie.

The visual sources and sounds were recombined using Lightworks movie editing software [24]. In our naming convention, the first capital letter indicates the valence of the sound (with a subscript s), and the second capital letter indicates the valence of the video (with a subscript v). When we recombined the original unpleasant audio and video tracks, we produced unpleasant *movies* (*U*_*s*_*U*_*v*_). Hereafter, “video track” refers to the visual event depicted in a movie, whereas “movie” refers to a combined auditory and visual stimulus denoted by two letters. Next, the unpleasant sounds were paired with the video track depicting a similarly timed neutral visual source (i.e., *U*_*s*_*N*_*v*_). In this manner, we created 20 *U*_*s*_*U*_*v*_ and 20 *U*_*s*_*N*_*v*_ movies. We also used two *U*_*s*_*U*_*v*_ and two *U*_*s*_*N*_*v*_ movies from Samermit and colleagues [22]: *person sipping through a straw*, and *person tapping fingers on table*. We note that our term *U*_*s*_*N*_*v*_ corresponds to the PAVS term used by Samermit et al. [16,23]; however, it was necessary to create different terminology to encompass our greater variety of stimulus conditions, which included video tracks, audio tracks, images, and text descriptions (see Table 1 for terms). Matching capital letters such as *UU* imply an original movie whereas mismatching letters such as *UN* imply that a sound was paired with a stimulus of a different valence. Next, for use in Experiment 3A, 22 complementary movies were made from films of neutral events that produced *N*_*v*_. We produced neutral movies (*N*_*s*_*N*_*v*_) and movies in which the neutral sound was paired with the corresponding unpleasant visual source of a trigger sound (i.e., *N*_*s*_*U*_*v*_). Because the two *U*_*s*_*N*_*v*_ movies from Samermit et al. [16,23] (a *stream flowing* and *person bouncing a ball on table*) did not contain the original neutral sounds, we made Foley recordings for those video tracks to create two corresponding *N*_*s*_*N*_*v*_ movies. This process resulted in a total of 44 movies available in a public repository (**https://doi.org/10.1184/R1/c.7112221**).

**Table 1:** Experiments with Unpleasant (*U_s_*) or Neutral (*N_s_*) audio tracks, Unpleasant (*U_v_*) or Neutral (*N_v_*) video tracks, Unpleasant (*U_D_*) or Neutral (*N_D_*) written descriptions, or Pleasant paintings (*P_p_*).

Written Description of Sources		Experiment 1		Experiment 2		Experiment 3A		Experiment 5A		Experiment 5B	
		**Order A**		**Order A**		**Order A**					
		(Order B = swap first and second half of Order A)									
**Unpleasant source**	**Alternative Neutral source**	**First Half**	**Second Half**	**First Half**	**Second Half**	**First Half**	**Second Half**	**First Half**	**Second Half**	**First Half**	**Second Half**
Person smacking their lips	Person pulling tape on and off tape dispenser	*U*_*s*_ *U*_*v*_	*U*_*s*_ *N*_*v*_	*U*_*s*_ *U*_*D*_	*U*_*s*_ *N*_*D*_	*N*_*s*_ *N*_*v*_	*N*_*s*_ *U*_*v*_	*U*_*s*_	*U*_*s*_ *P*_*p*_	*U*_*s*_	*U*_*s*_ *N*_*v*_
Person brushing their teeth	Lawn sprinkler spraying water	*U*_*s*_ *N*_*v*_	*U*_*s*_ *U*_*v*_	*U*_*s*_ *N*_*D*_	*U*_*s*_ *U*_*D*_	*N*_*s*_ *U*_*v*_	*N*_*s*_ *N*_*v*_	*U*_*s*_	*U*_*s*_ *P*_*p*_	*U*_*s*_	*U*_*s*_ *N*_*v*_
Person eating chips	Person shaking a bottle containing beads	*U*_*s*_ *U*_*v*_	*U*_*s*_ *N*_*v*_	*U*_*s*_ *U*_*D*_	*U*_*s*_ *N*_*D*_	*N*_*s*_ *N*_*v*_	*N*_*s*_ *U*_*v*_	*U*_*s*_	*U*_*s*_ *P*_*p*_	*U*_*s*_	*U*_*s*_ *N*_*v*_
Person crinkling a plastic bottle	Campfire burning	*U*_*s*_ *N*_*v*_	*U*_*s*_ *U*_*v*_	*U*_*s*_ *N*_*D*_	*U*_*s*_ *U*_*D*_	*N*_*s*_ *U*_*v*_	*N*_*s*_ *N*_*v*_	*U*_*s*_	*U*_*s*_ *P*_*p*_	*U*_*s*_	*U*_*s*_ *N*_*v*_
Person cracking their knuckles	Person snapping a stick	*U*_*s*_ *N*_*v*_	*U*_*s*_ *U*_*v*_	*U*_*s*_ *N*_*D*_	*U*_*s*_ *U*_*D*_	*N*_*s*_ *U*_*v*_	*N*_*s*_ *N*_*v*_	*U*_*s*_	*U*_*s*_ *P*_*p*_	*U*_*s*_	*U*_*s*_ *N*_*v*_
Person sniffing 1	Person scraping a ruler on a table	*U*_*s*_ *N*_*v*_	*U*_*s*_ *U*_*v*_	*U*_*s*_ *N*_*D*_	*U*_*s*_ *U*_*D*_	*N*_*s*_ *U*_*v*_	*N*_*s*_ *N*_*v*_	*U*_*s*_	*U*_*s*_ *P*_*p*_	*U*_*s*_	*U*_*s*_ *N*_*v*_
Person scraping a fork and knife together	Birds chirping	*U*_*s*_ *U*_*v*_	*U*_*s*_ *N*_*v*_	*U*_*s*_ *U*_*D*_	*U*_*s*_ *N*_*D*_	*N*_*s*_ *N*_*v*_	*N*_*s*_ *U*_*v*_	*U*_*s*_	*U*_*s*_ *P*_*p*_	*U*_*s*_	*U*_*s*_ *N*_*v*_
Person sniffing 2	Person pulling facial tissues out of a box	*U*_*s*_ *U*_*v*_	*U*_*s*_ *N*_*v*_	*U*_*s*_ *U*_*D*_	*U*_*s*_ *N*_*D*_	*N*_*s*_ *N*_*v*_	*N*_*s*_ *U*_*v*_	*U*_*s*_	*U*_*s*_ *P*_*p*_	*U*_*s*_	*U*_*s*_ *N*_*v*_
Person typing on a keyboard	Person twisting a Rubik’s cube	*U*_*s*_ *U*_*v*_	*U*_*s*_ *N*_*v*_	*U*_*s*_ *U*_*D*_	*U*_*s*_ *N*_*D*_	*N*_*s*_ *N*_*v*_	*N*_*s*_ *U*_*v*_	*U*_*s*_	*U*_*s*_ *P*_*p*_	*U*_*s*_	*U*_*s*_ *N*_*v*_
Person sucking air in through their teeth	Person pulling and releasing measuring tape	*U*_*s*_ *N*_*v*_	*U*_*s*_ *U*_*v*_	*U*_*s*_ *N*_*D*_	*U*_*s*_ *U*_*D*_	*N*_*s*_ *U*_*v*_	*N*_*s*_ *N*_*v*_	*U*_*s*_	*U*_*s*_ *P*_*p*_	*U*_*s*_	*U*_*s*_ *N*_*v*_
Person coughing	Person tapping a bag that is laying on top of a tambourine	*U*_*s*_ *U*_*v*_	*U*_*s*_ *N*_*v*_	*U*_*s*_ *U*_*D*_	*U*_*s*_ *N*_*D*_	*N*_*s*_ *N*_*v*_	*N*_*s*_ *U*_*v*_	*U*_*s*_	*U*_*s*_ *P*_*p*_	*U*_*s*_	*U*_*s*_ *N*_*v*_
Person chewing gum	Person stirring noodle soup	*U*_*s*_ *N*_*v*_	*U*_*s*_ *U*_*v*_	*U*_*s*_ *N*_*D*_	*U*_*s*_ *U*_*D*_	*N*_*s*_ *U*_*v*_	*N*_*s*_ *N*_*v*_	*U*_*s*_	*U*_*s*_ *P*_*p*_	*U*_*s*_	*U*_*s*_ *N*_*v*_
Person swishing water in their mouth	Duck splashing in water basin	*U*_*s*_ *N*_*v*_	*U*_*s*_ *U*_*v*_	*U*_*s*_ *N*_*D*_	*U*_*s*_ *U*_*D*_	*N*_*s*_ *U*_*v*_	*N*_*s*_ *N*_*v*_	*U*_*s*_	*U*_*s*_ *P*_*p*_	*U*_*s*_	*U*_*s*_ *N*_*v*_
Person scratching their scalp	Deer eating leaves	*U*_*s*_ *U*_*v*_	*U*_*s*_ *N*_*v*_	*U*_*s*_ *U*_*D*_	*U*_*s*_ *N*_*D*_	*N*_*s*_ *N*_*v*_	*N*_*s*_ *U*_*v*_	*U*_*s*_	*U*_*s*_ *P*_*p*_	*U*_*s*_	*U*_*s*_ *N*_*v*_
Person gulping water	Bubbles rising in watercooler	*U*_*s*_ *N*_*v*_	*U*_*s*_ *U*_*v*_	*U*_*s*_ *N*_*D*_	*U*_*s*_ *U*_*D*_	*N*_*s*_ *U*_*v*_	*N*_*s*_ *N*_*v*_	*U*_*s*_	*U*_*s*_ *P*_*p*_	*U*_*s*_	*U*_*s*_ *N*_*v*_
Person wheezing	Person pressing an air pump	*U*_*s*_ *U*_*v*_	*U*_*s*_ *N*_*v*_	*U*_*s*_ *U*_*D*_	*U*_*s*_ *N*_*D*_	*N*_*s*_ *N*_*v*_	*N*_*s*_ *U*_*v*_	*U*_*s*_	*U*_*s*_ *P*_*p*_	*U*_*s*_	*U*_*s*_ *N*_*v*_
Person breathing noisily	Person dragging a dust broom across table	*U*_*s*_ *U*_*v*_	*U*_*s*_ *N*_*v*_	*U*_*s*_ *U*_*D*_	*U*_*s*_ *N*_*D*_	*N*_*s*_ *N*_*v*_	*N*_*s*_ *U*_*v*_	*U*_*s*_	*U*_*s*_ *P*_*p*_	*U*_*s*_	*U*_*s*_ *N*_*v*_
Person sneezing	Person spraying water with spray bottle	*U*_*s*_ *N*_*v*_	*U*_*s*_ *U*_*v*_	*U*_*s*_ *N*_*D*_	*U*_*s*_ *U*_*D*_	*N*_*s*_ *U*_*v*_	*N*_*s*_ *N*_*v*_	*U*_*s*_	*U*_*s*_ *P*_*p*_	*U*_*s*_	*U*_*s*_ *N*_*v*_
Person scratching a blackboard	Person ripping fabric	*U*_*s*_ *N*_*v*_	*U*_*s*_ *U*_*v*_	*U*_*s*_ *N*_*D*_	*U*_*s*_ *U*_*D*_	*N*_*s*_ *U*_*v*_	*N*_*s*_ *N*_*v*_	*U*_*s*_	*U*_*s*_ *P*_*p*_	*U*_*s*_	*U*_*s*_ *N*_*v*_
Person blowing their nose	Person releasing air from a balloon	*U*_*s*_ *U*_*v*_	*U*_*s*_ *N*_*v*_	*U*_*s*_ *U*_*D*_	*U*_*s*_ *N*_*D*_	*N*_*s*_ *N*_*v*_	*N*_*s*_ *U*_*v*_	*U*_*s*_	*U*_*s*_ *P*_*p*_	*U*_*s*_	*U*_*s*_ *N*_*v*_
Person sipping through a straw [Samermit et al., 2022; Vid01_O]	Stream flowing [Samermit et al., 2022; Vid01_P]	*U*_*s*_ *N*_*v*_	*U*_*s*_ *U*_*v*_	*U*_*s*_ *N*_*D*_	*U*_*s*_ *U*_*D*_	*N*_*s*_ *U*_*v*_	*N*_*s*_ *N*_*v*_	*U*_*s*_	*U*_*s*_ *P*_*p*_	*U*_*s*_	*U*_*s*_ *N*_*v*_
Person tapping on a table [Samermit et al., 2022; Vid03_O]	Person bouncing a ball on table [Samermit et al., 2022; Vid03_P]	*U*_*s*_ *U*_*v*_	*U*_*s*_ *N*_*v*_	*U*_*s*_ *U*_*D*_	*U*_*s*_ *N*_*D*_	*N*_*s*_ *N*_*v*_	*N*_*s*_ *U*_*v*_	*U*_*s*_	*U*_*s*_ *P*_*p*_	*U*_*s*_	*U*_*s*_ *N*_*v*_

This only lists studies that used different stimuli in their first and second halves.

As part of stimulus development, we measured the baseline pleasantness ratings for individual silent video tracks and audio tracks; see Supplementary File S1 for method details and Supplementary File S2 (“Baseline video pleasantness” and “Experiment 4”) for pleasantness ratings.

#### Participant populations.

Participant recruitment began on 01/07/2022 and ended on 23/04/2024. We recruited participants online through Carnegie Mellon University system and through Prolific [30]. At the time of recruitment, all participants completed one or more of the following questionnaires that assessed misophonia severity: MisoQuest [31], Misophonia Questionnaire (MQ) [32], Duke-Vanderbilt Misophonia Screening Questionnaire (DVMSQ) [33] and the S-Five [34]. We followed the scoring guidelines for each questionnaire to determine misophonic severity for each individual. For Experiments 1 and 2, using the tabulated scores for each individual, we categorized listeners into one of two groups: a misophonic or non-misophonic group. The misophonic group included all participants who received a subclinical or clinical misophonia score on any of the questionnaires. This umbrella criterion for misophonia [35] includes people who experience severe “misophonic reactions” without requiring that they also have a clinically relevant functional impairment in their daily lives. Note, additional recruitment for misophonic participants was conducted for individuals in the same age range as our non-misophonic population through Prolific, flyers posted in the Pittsburgh region, soQuiet.org [36], and social media (e.g., Facebook, Reddit). The non-misophonic control group included participants who received a nonclinical score on all questionnaires. For the remaining experiments (i.e., Experiments 3, 4, 5), the participants were recruited irrespective of their misophonic severity. We refer to these participants as our *unscreened* group, reflecting a subset of the population in which *some* individuals may have high misophonic severity. All *unscreened* participants were included in one group for data analyses. In contrast, for data analyses in Experiments 1 and 2, we compared misophonic and non-misophonic groups. Thus, our non-misophonic control group is not equivalent to our *unscreened* group because the *unscreened* group may contain some misophonic participants. Across all *unscreened* groups (i.e., Experiments 3A, 4 and 5A-B; *N* = 154), the percentage of individuals who received a clinical or subclinical misophonia score was 11.69%, or 9.09%, respectively. Note that participant recruitment for all *unscreened* groups (Expts. 3–5) described the study as being about “judging properties of sounds and/or videos” and did not mention misophonia or unpleasant sounds.

#### Common procedures.

Normal hearing and corrected-to-normal vision were required. First, in all studies, participants gave written consent through an online form approved by Carnegie Mellon’s Institutional Review Board (IRB#2015_00000409). Prior to completing the experimental trials online using Qualtrics [37], each participant answered questions about their age, gender, and vision/hearing status. In some cases, optional questions about ethnicity were recorded (see S2 Table). Subsequently, participants completed a volume calibration to ensure that all sounds and movies were played at a comfortably audible level. Next, participants completed a binaural Huggins’ pitch test [38] to verify that they were wearing a pair of quality headphones. If participants passed the headphone screening, they completed questionnaires that assessed misophonia severity. Next, participants completed one practice trial (a replica of a real trial in the upcoming condition) to orient them to the question format. Within the experimental trials, a catch-trial was implemented to ensure that participants were fully attentive throughout the duration of the study. This catch trial appeared superficially like the other trials, but the instructions were different. We excluded all responses from participants who failed to correctly answer the catch-trial. Additionally, we examined data from participants to see if they provided only one or two values for all ratings, but no problematic cases were found after the headphone screening and catch-trial criteria were applied. Numbers of excluded participants are reported within the methods section of each individual study. Experimental trials took approximately 30 minutes per study. Participants were compensated for their time with money or credit to fulfill a course requirement at CMU. In all studies involving ratings of sounds, participants were asked “How pleasant is the sound?” before selecting a response from an 11-point scale, wherein -5 indicated the sound was very unpleasant, +5 indicated the sound was very pleasant, and 0 indicated the sound was neutral [8].

## Experiment 1: Altering the pleasantness of an unpleasant sound with neutral or unpleasant visual sources

We predicted that our unpleasant sounds would be more pleasant when paired with an alternative neutral visual source (*U*_*s*_*N*_*v*_) than when paired with a visual source that depicted the true cause of the sound (*U*_*s*_*U*_*v*_). To test the effectiveness of the movies we created on a misophonic population, we implemented a study design in which every trial contains a different movie, with no repetition of sounds in the first half of the experiment. Within the first half of the experiment, half the trials are *U*_*s*_*N*_*v*_ and the other half are *U*_*s*_*U*_*v*_. This design allows us to measure the pleasantness of a sound upon its first exposure when it is accompanied by a visual source. The complementary visual sources are shown in the second half, which is a second exposure to each sound. By always accompanying the sound with a visual source, we can ensure the intended identification of the sound’s source in each condition, minimizing the effects of any sound ambiguity. We compare a misophonic and non-misophonic group. We examine the relationship between audiovisual match and movie effectiveness to test the *source reassignment hypothesis*.

### Method

#### Participants.

Eighty-two participants (M_age_ = 24.5 years; range = 18–36 years; 42 females, 38 males, two non-binary) were tested (after excluding 23 and 18 participants for failing catch trials and headphone screening, respectively). In total, 20 participants (M_age_ = 24.75 years; range = 18–36 years; 8 females, 10 males, two non-binary) met our criterion for misophonia (see General Methods).

#### Stimuli.

The 22 unpleasant sounds, *U*_*s*_, combined with a video of an alternative neutral visual source, *N*_*v*_ to produce a movie, *U*_*s*_*N*_*v*_. This process created 22 movies (see General Methods). Additionally, the unpleasant sounds were combined with their original visual sources, *U*_*s*_*U*_*v*_. Our total stimulus set was 44 movies, divided equally amongst the two conditions (see Procedure below, and Table 1).

#### Procedure.

Each of the 44 trials contained a unique movie. Participants saw every movie and were randomly assigned to watch them in one of two presentation orders (see Table 1). Within each order, there were two mutually exclusive presentation halves. In the first half of order A, all 22 sounds were used in 22 movies: 11 sounds were in *U*_*s*_*U*_*v*_ pairs, and the remaining 11 sounds were in *U*_*s*_*N*_*v*_ pairs. In the second half of order A, the complementary 11 *U*_*s*_*N*_*v*_ pairs and 11 *U*_*s*_*U*_*v*_ pairs were presented (see Table 1). In order B, the second half of order A was presented first. For example, in the first half of order A, the sound of a *person smacking their lips* was paired with an unpleasant visual source (*U*_*s*_*U*_*v*_), while in the second half of order A, it was paired with a neutral visual source (*U*_*s*_*N*_*v*_). In the first half of order B, the sound of a *person smacking their lips* was paired with a neutral visual source (*U*_*s*_*N*_*v*_), while in the second half of order B, the same sound was paired with an unpleasant visual source (*U*_*s*_*U*_*v*_). By this design, in the first half of the study, every unpleasant sound was heard only once, and each sound was only paired with one visual source.

After observing a movie, participants rated the pleasantness of the sound within the movie. Specifically, participants were asked “How pleasant is the sound?” before selecting a response from an 11-point scale, wherein -5 indicated the sound was very unpleasant, +5 indicated the sound was very pleasant, and 0 indicated the sound was neutral [6]. Next, as in Samermit and colleagues [23], participants were asked to rate “How well does the sound match the visual event?” with a 5-point scale, for which 1 indicated “not a good match”, and 5 indicated an “extremely good match.” To clarify the meaning of “match,” we added that participants should rate “How likely it is that the visual sources caused the sounds to occur.” In our discussion, we will refer to this as the “plausibility” definition of match. The presentation order of the movies within their respective sections was random.

### Results

Experiment 1 tested the prediction that the unpleasant sounds would be rated as more pleasant when paired with an alternative neutral visual source (*U*_*s*_*N*_*v*_) than when paired with a visual source that depicted the true cause of the sound (*U*_*s*_*U*_*v*_). To assess differences in sound pleasantness ratings, we conducted a mixed-design ANOVA with repeated measures of visual source pairing (i.e., *U*_*s*_*N*_*v*_ or *U*_*s*_*U*_*v*_) and presentation half (first or second), and between-subjects factors of order (A or B), misophonic status (misophonic or non-misophonic), and gender. Age was not included as a factor because the misophonic and non-misophonic groups were similar in age. As expected, sound pleasantness ratings depended upon pairing (*F*(1, 73) = 80.123, *p* < 0.001, *η*_*p*_^*2*^ = 0.523, power = 1.0). On average, the sound pleasantness ratings were reliably lower for misophonics than non-misophonics (*F*(1, 73) = 9.615, *p* = 0.003, *η*_*p*_^*2*^ = 0.116, power = 0.864). There was an interaction between these two factors, indicating that the difference in sound pleasantness ratings between *U*_*s*_*N*_*v*_ and *U*_*s*_*U*_*v*_ pairs was reliably larger for misophonics compared to non-misophonics (*F*(1, 73) = 5.709, *p* = 0.019, *η*_*p*_^*2*^ = 0.073, power = 0.655). Furthermore, we did observe a main effect of gender with females giving lower ratings (*F*(2, 73) = 5.398, *p* = 0.007, *η*_*p*_^*2*^ = 0.129, power = 0.830), and an interaction between gender and misophonic status (*F*(1, 73) = 4.992, *p* = 0.029, *η*_*p*_^*2*^ = 0.064, power = 0.597) indicating that females with misophonia gave sounds the lowest pleasantness ratings. However, gender did not interact with any of the stimulus factors.

We did not observe a main effect of presentation half (*F*(1, 73) = 0.143, *p* = 0.707, *η*_*p*_^*2*^ = 0.002, power = 0.066) nor order (*F*(1, 73) = 1.470, *p* = 0.229, *η*_*p*_^*2*^ = 0.020, power = 0.223), nor an interaction between these two factors. We did observe a three-way interaction between order, visual pairing, and presentation half (*F*(1, 73) = 9.741, *p* = 0.003, *η*_*p*_^*2*^ = 0.118, power = 0.869). This interaction suggests that the size of the difference between *U*_*s*_*N*_*v*_ and *U*_*s*_*U*_*v*_ pairs was, on average, smaller in the second half (as found in Samermit et al. [23]), but this pattern depended on the test order (A or B). Because of the interaction between presentation half and test order, our subsequent analyses and figures draw only from data in the first half of the study, i.e., the first exposure to each sound.

Analyzing only the first presentation effectively transforms our study into a between-subjects design, which means that each data point in subsequent figures represents a movie that was rated by 41 of the 82 participants. The average sound pleasantness rating for misophonics in the first half of the study, taken across all 22 sounds in the *U*_*s*_*U*_*v*_ pairing, -2.21 (SD = 1.03), was significantly lower than the average sound pleasantness rating in the *U*_*s*_*N*_*v*_ pairing, -0.63 (SD = 1.52) (*t*(21) = 5.91, *p* < 0.001). This was also true for non-misophonics (M_*UsUv*_ = -1.32, SD = 0.92; M_*UsNv*_ = -0.34, SD = 1.07) (*t*(21) = 4.65, *p* < 0.001). The misophonics rated *U*_*s*_*U*_*v*_ pairs as having lower pleasantness than non-misophonics (*t*(42) = -3.03, *p* < 0.004); however, they did not provide significantly lower pleasantness ratings than non-misophonics for *U*_*s*_*N*_*v*_ pairs. The change in pleasantness due to visual pairing was marginally greater for misophonics (*t*(40) = 1.78, *p* = 0.082). The average match quality rating for misophonics of *U*_*s*_*N*_*v*_ pairs was 2.80 (SD = 1.05) on a scale of 1–5 with a range from 1.00 to 4.70, while the average match quality of *U*_*s*_*U*_*v*_ pairs was 4.00 (SD = 0.53) with a range from 2.40 to 4.60. The average match quality for non-misophonics of *U*_*s*_*N*_*v*_ pairs was 2.88 (SD = 0.73) with a range from 1.52 to 3.94, while the average match quality of *U*_*s*_*U*_*v*_ pairs was 3.63 (SD = 0.48) with a range from 2.26 to 4.13. The misophonics rated *U*_*s*_*U*_*v*_ pairs as having higher match quality than non-misophonics (*t*(42) = 2.44, *p* = 0.019); however, misophonics did not provide higher match quality ratings than non-misophonics for *U*_*s*_*N*_*v*_ pairs. The relationship between average sound pleasantness of sound-visual pairs versus their respective match quality rating is illustrated in Supplemental S1 and S2 Figs for our two populations. More pleasant sound-visual pairs were significantly associated with higher match quality ratings for *U*_*s*_*N*_*v*_ pairs (Misophonics: R^2^ = 0.41, *F*(1, 20) = 13.94, *p* = 0.001; Non-misophonics: R^2^ = 0.61, *F*(1, 20) = 30.95, *p* < 0.001), but no such association was seen for *U*_*s*_*U*_*v*_ pairs (Misophonics: R^2^ = 0.04, *F*(1, 20) = 0.85, *p* = 0.37; Non-misophonics: R^2^ = 0.02, *F*(1, 20) = 0.42, *p* = 0.53).

**Fig 1:**
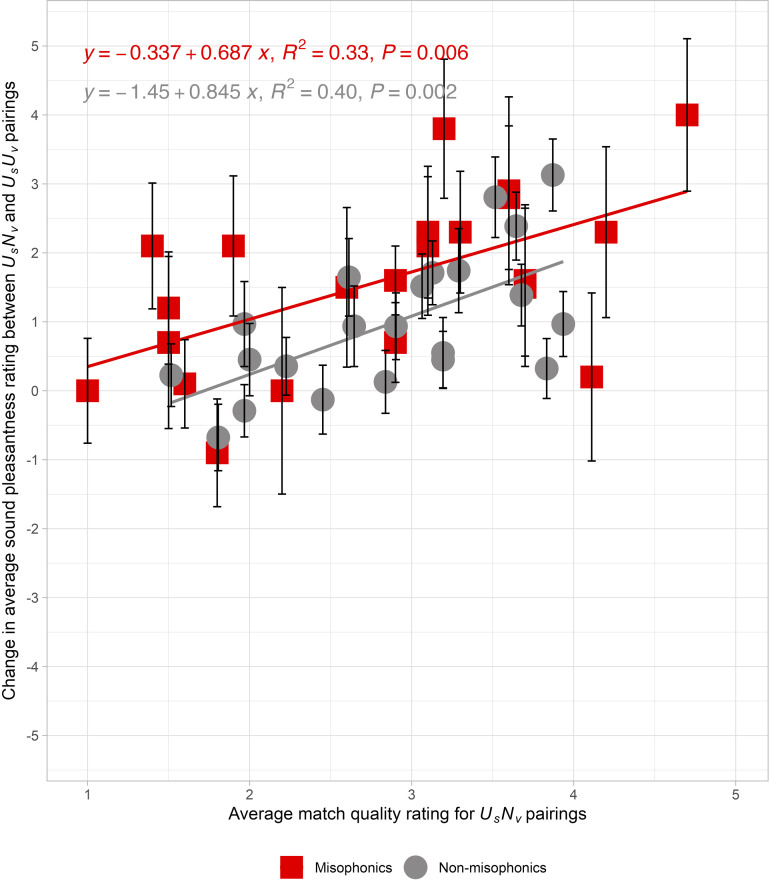
Experiment 1: Unpleasant sounds paired with neutral or unpleasant visual sources. The relationship between the change in average sound pleasantness ratings across the neutral (*U*_*s*_*N*_*v*_) and unpleasant (*U*_*s*_*U*_*v*_) pairs versus the average match quality ratings for *U*_*s*_*N*_*v*_ pairs in Experiment 1. Changes were calculated by subtracting the average pleasantness rating of *U*_*s*_*U*_*v*_ from *U*_*s*_*N*_*v*_. The averages were computed within two mutually exclusive participant groups: Misophonics (red squares), and Non-misophonics (gray circles). The solid line represents the linear regression fit to the data. Each data point reflects the mean change for each unpleasant sound, with error bars reflecting the standard error of the mean across participants.

Fig 1, displaying data from the first presentation half of Experiment 1, depicts a *change function*: the subtraction of the average sound pleasantness rating (*U*_*s*_*U*_*v*_ - *U*_*s*_*N*_*v*_) as a function of the match quality rating of *U*_*s*_*N*_*v*_ pairs. The average sound pleasantness ratings for misophonics (*N* = 20) are represented by red squares, while the average sound pleasantness rating for non-misophonics (N = 62) are represented by gray circles. For misophonics, an increase in match quality of 1 point for *U*_*s*_*N*_*v*_ pairing is associated with a benefit of 0.69 pleasantness rating points for sounds in *U*_*s*_*N*_*v*_ pairs over the *U*_*s*_*U*_*v*_ pairs (R^2^ = 0.33, *F*(1, 20) = 9.67, *p* = 0.006). At the lowest match quality rating (1), the change in sound pleasantness was approximately 0.35 points whereas at the highest match quality rating (5), the change in sound pleasantness is projected to be 3.40 points. We observe that 19 of the 22 data points on the *change function* are above zero, showing a pleasantness benefit from a neutral relative to an unpleasant visual source. In particular, the sounds with the largest pleasantness change for misophonics were: *person brushing their teeth, person swishing water in their mouth, and person eating chips*, which changed in pleasantness by 4.00, 3.80, and 2.90 points, respectively. For non-misophonics, an increase in match quality of 1 point for *U*_*s*_*N*_*v*_ pairing is associated with a benefit of 0.85 pleasantness rating points for sounds in *U*_*s*_*N*_*v*_ pairs over the *U*_*s*_*U*_*v*_ pairs (R^2^ = 0.40, *F*(1, 20) = 13.08, *p* = 0.002). At the lowest match quality rating (1), the change in sound pleasantness was approximately -0.61points whereas at the highest match quality rating (5), the change in sound pleasantness is projected to be 2.77 points. Again, we observed that 19 of the 22 data points on the *change function* are above zero. The sounds with the largest pleasantness changes for non-misophonics were: *person brushing their teeth, person swishing water in their mouth*, and *person crinkling a plastic bottle*, which changed in pleasantness by 3.12, 2.81, and 2.39 points, respectively. Supplemental S3 Fig depicts a non-significant, horizontal *change function* across the match quality rating of *U*_*s*_*U*_*v*_ pairs for both populations (Misophonics: R^2^ = 0.04, *F*(1, 20) = 0.81, *p* = 0.38; Non-misophonics: R^2^ = 0.06, *F*(1, 20) = 1.37, *p* = 0.26), which provides evidence that the video sources which matched their sounds were not driving the source reassignment. In sum, the change in pleasantness due to visual pairs is related to the match quality of the *U*_*s*_*N*_*v*_ movies that reassign the source, and this change is larger for the misophonic participants.

To assess the quality of our stimuli, we plotted our results from Fig 1 alongside those of Samermit et al. [23] in Supplemental S4 Fig. We used their published supplemental data to compute a *change function* for their stimuli. The data points from our two studies fall along nearly identical regression lines and the stimuli cover similar ranges of match and pleasantness ratings. This close quantitative replication of a prior study validates the effectiveness of our new movies and illustrates the generalizability of match ratings as a predictor of the effectiveness of differing stimuli.

Finally, we looked for evidence that the pleasantness of the visual sources was influencing the sound ratings. However, the difference in pleasantness of the individual silent visual sources (*U*_*v*_ - *N*_*v*_) did not correlate with the change in average sound pleasantness (*U*_*s*_*N*_*v-*_ - *U*_*s*_*U*_*v*_) in Experiment 1 (*r* = -0.26, R^2^ = 0.07, *F*(1, 20) = 1.47, *p* = 0.238). Therefore, there is no evidence that participants were rating visual pleasantness instead of rating the sound pleasantness.

## Experiment 2: Altering the pleasantness of an unpleasant sound with written descriptions of neutral or unpleasant sources

Experiment 1 implies that movies can change a sound’s pleasantness by changing its attributed source. Experiment 2 tested the prediction that this same effect could be accomplished without movies. Text descriptions of the visual sources used in Experiment 1 were used in place of the video tracks. If the underlying effect of the movies is source reassignment, and if text descriptions semantically convey the same sources that movies provide, this study should show good strong agreement with Experiment 1. To the extent that the text descriptions are less convincing than movies, this study should show a smaller effect size than the comparable movie study. As in Experiment 1, we tested both misophonic and non-misophonic groups.

### Method

#### Participants.

Eighty-one participants (M_age_ = 22.04 years; range = 18–30 years; 41 females, 34 males, five non-binary and one prefer not to say) were tested (after excluding 3 and 39 participants for failing catch trials and headphone screening, respectively). In total, 26 participants (M_age_ = 22.27 years; range = 18–29 years; 16 females, 6 males, four non-binary) met our criteria for misophonia. Note, there were 21 individuals who did not complete the second half of the study due to time constraints. Their data were removed from our omnibus ANOVA but were included in our analysis of the first half of the study.

#### Stimuli.

The 22 *U*_*s*_ were combined with a text description of the cause of the sound. The text descriptions of the cause of the sound either matched its original source (i.e., an unpleasant sound paired with its true, unpleasant description, *U*_*s*_*U*_*D*_), or matched the neutral visual source (i.e., an unpleasant sound paired with an alternative neutral description, *U*_*s*_*N*_*D*_). The descriptions contained enough information for the listener to get a sense of the source event but lacked significant detail. For example, the text description for the trigger sound of crunchy chewing was “*Person eating chips*” while the text description of its neutral counterpart was “*Person shaking a bottle containing beads.*” See Table 1 for descriptions of all 44 videos.

#### Procedure.

Participants completed the same sequence of experimental procedures as outlined in Experiment 1. Instead of watching short movies, participants were told that they would be listening to a short sound accompanied by a text description of its cause. As in Experiment 1, they were instructed to judge sound pleasantness and match quality. Half the participants were tested in each test order.

### Results

Experiment 2 tested the prediction that the pleasantness ratings of unpleasant sounds would be higher when paired with a text description that offered a neutral cause of the sound (*U*_*s*_*N*_*D*_) compared to the original, unpleasant cause of the sound (*U*_*s*_*U*_*D*_). We conducted a mixed-design ANOVA with repeated measures of description pairing (*N*_*D*_ or *U*_*D*_) and presentation half (first or second) and between-subject factors of order (A or B), misophonic status (misophonic or non-misophonic) and gender. Sound pleasantness ratings for unpleasant sounds depended upon the pairing of the neutral or unpleasant description (*F*(1, 48) = 52.640, *p* < 0.001, *η*_*p*_^*2*^ = 0.523, power = 1.0). On average, the sound pleasantness ratings were reliably lower for misophonics compared to non-misophonics (*F*(1, 48) = 27.920, *p* < 0.001, *η*_*p*_^*2*^ = 0.368, power = 1.0). The mean change in sound pleasantness ratings between *U*_*s*_*N*_*D*_ and *U*_*s*_*U*_*D*_ pairs was larger for misophonics compared to non-misophonics; however, this difference was not significant (*F*(1, 48) = 2.383, *p* = 0.129, *η*_*p*_^*2*^ = 0.047, power = 0.328). We did not observe a main effect of gender (*F*(1, 48) = 2.579, *p* = 0.115, *η*_*p*_^*2*^ = 0.051, power = 0.350), nor an interaction between gender and misophonic status (*F*(1, 48) = 2.542, *p* = 0.117, *η*_*p*_^*2*^ = 0.050, power = 0.346), nor any of the stimulus level factors. There was no main effect or interaction for the presentation half or order.

As in Experiment 1, the remainder of our analyses exclusively use the responses from the first time each unpleasant sound was heard. The average sound pleasantness rating for misophonics, taken across all 22 sounds in the *U*_*s*_*U*_*D*_ pairing, -2.40 (SD = 1.14), was significantly lower than the average sound pleasantness rating in the *U*_*s*_*N*_*D*_ pairing, -1.43 (SD = 1.78) (*t*(21) = 3.75, *p* = 0.001). This was also true for non-misophonics (M_*UsUD*_ = -1.38, SD = 1.52; M_*UsND*_ = -0.85, SD = 1.57) (*t*(21) = 2.73, *p* = 0.013). The misophonics rated *U*_*s*_*U*_*D*_ pairs as having lower pleasantness than non-misophonics (*t*(42) = -2.50, *p* <0.02); however, they did not provide significantly lower pleasantness ratings than non-misophonics for *U*_*s*_*N*_*D*_ pairs. The pleasantness change due to description pairing was marginally larger for misophonics (M = 0.97, SD = 1.21) than non-misophonics (M = 0.53, SD = 0.91) (*t*(21) = 1.81, *p* = 0.085). For misophonics, the average match quality rating of *U*_*s*_*N*_*D*_ pairs was 2.36 (SD = 0.78) with a range from 1.00 to 3.90, while the average match quality of *U*_*s*_*U*_*D*_ pairs was 3.86 (SD = 0.59) with a range from 2.33 to 4.72. For non-misophonics, the average match quality of *U*_*s*_*N*_*D*_ pairs was 2.23 (SD = 0.66) with a range from 1.08 to 3.48, while the average match quality of *U*_*s*_*U*_*D*_ pairs was 3.62 (SD = 0.67) with a range from 1.85 to 4.72. The match quality ratings did not differ depending on misophonic status for *U*_*s*_*N*_*D*_ pairs (*t*(41) = 0.63, *p* = 0.53), nor *U*_*s*_*U*_*D*_ pairs (*t*(41) = 1.24, *p* = 0.22). The relationship between average sound pleasantness of sound-description pairs versus their respective match quality rating is illustrated in Supplemental S5 and S6 Figs for both populations. For both groups, higher sound pleasantness was significantly associated with higher match quality ratings for *U*_*s*_*N*_*D*_ pairs (Misophonics: R^2^ = 0.48, *F*(1, 20) = 18.38, *p* < 0.001; Non-misophonics: R^2^ = 0.48, *F*(1, 20) = 18.55, *p* < 0.001), but there was no such association for *U*_*s*_*U*_*D*_ pairs (Misophonics: R^2^ = 0.04, *F*(1, 20) = 0.91, *p* = 0.35; Non-misophonics: R^2^ = 0.06, *F*(1, 20) = 1.26, *p* = 0.28).

**Fig 2:**
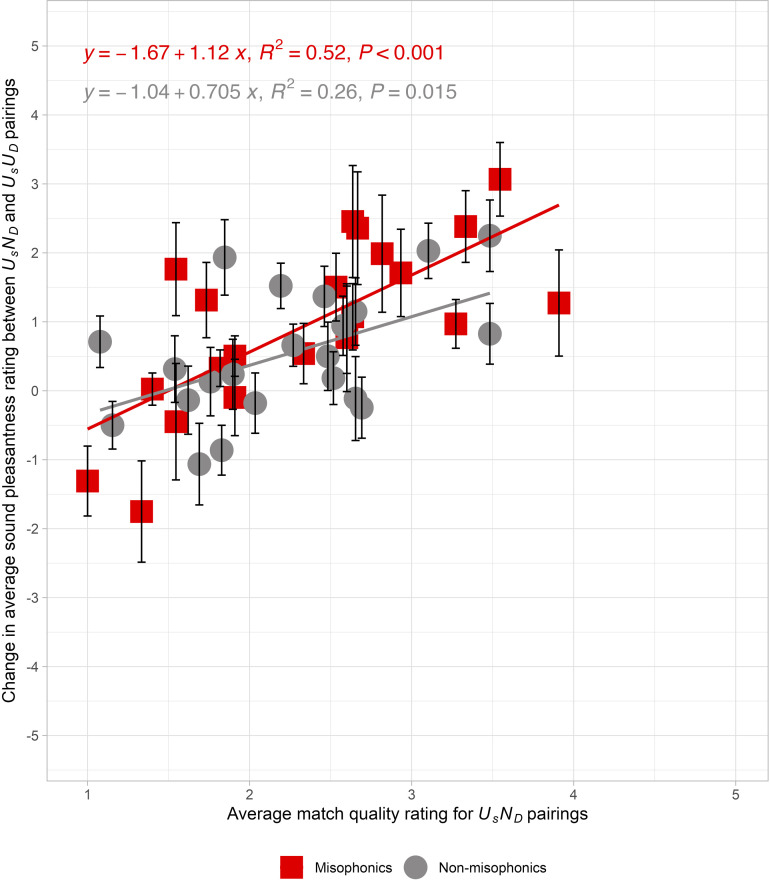
Experiment 2: Unpleasant sounds paired with neutral or unpleasant event descriptions. The relationship between the change in average sound pleasantness ratings across the neutral (*U*_*s*_*N*_*D*_) and unpleasant (*U*_*s*_*U*_*D*_) pairs versus the average match quality ratings for *U*_*s*_*N*_*D*_ pairs in Experiment 2. The changes are calculated by subtracting the average pleasantness rating of *U*_*s*_*U*_*D*_ from *U*_*s*_*N*_*D*_. The averages are calculated within two mutually exclusive participant groups: Misophonics (red squares), and Non-misophonics (gray circles). The solid line indicates the linear regression fit to the data. Each data point represents the mean change for each of the unpleasant sounds and the error bars reflect the standard error of the mean across participants.

To illustrate the changes caused by description pairing, Fig 2 depicts a *change function*: the subtraction of average sound pleasantness rating (*U*_*s*_*U*_*D*_ - *U*_*s*_*N*_*D*_) pairs versus the match quality rating of *U*_*s*_*N*_*D*_ pairs. The average change in sound pleasantness ratings for the misophonics (N = 26) and non-misophonics (N = 55) are represented by red squares and gray circles, respectively. For misophonics, an increase in match quality of 1 point for *U*_*s*_*N*_*D*_ pairing is associated with an increase of 1.12 pleasantness rating points between *U*_*s*_*U*_*D*_ and *U*_*s*_*N*_*D*_ pairs (R^2^ = 0.52, *F*(1, 20) = 21.47, *p* < 0.001). At the lowest match quality rating (1), the *change function* is at -0.55 while at the highest match quality rating (5), the *change function* is projected to be at 3.91. For misophonics, 18 data points on the misophonic *change functions* are positive (i.e., a positive change from a neutral description). In particular, the sounds with the largest pleasantness change for misophonics were: *person scratching a blackboard, person crinkling a plastic bottle,* and *person typing on a keyboard*, which changed in pleasantness by 3.06, 2.46, and 2.38 points, respectively. For non-misophonics, we observe that an increase in match quality of 1 point for *U*_*s*_*N*_*v*_ pairing is associated with a pleasantness increase of 0.71 points between *U*_*s*_*U*_*D*_ and *U*_*s*_*N*_*D*_ pairs (R^2^ = 0.25, *F*(1, 20) = 7.00, *p* = 0.015). At the lowest match quality rating (1), the change in sound pleasantness is approximately -0.34 while at a high match quality rating (5), the change in sound pleasantness is projected to be to be 2.49. We observe that 15 of the 22 data points on the non-misophonic *change function* are positive. The sounds with the largest pleasantness change for non-misophonics were: *person scratching a blackboard, person swishing water in their mouth,* and *person scratching scalp*, which changed in pleasantness by 2.25, 2.03, and 1.93 points, respectively. Supplemental S7 Fig depicts a non-significant, horizontal *change function* across the match quality rating of *U*_*s*_*U*_*D*_ pairs for both populations (Misophonics: R^2^ = 0.07, *F*(1, 20) = 1.47, *p* = 0.24; Non-misophonics: R^2^ = 0.10, *F*(1, 20) = 2.19, *p* = 0.15), confirming that the match of the alternative source in the *U*_*s*_*N*_*D*_ pairing (in Fig 2) is what causes the change in sound pleasantness.

Comparing the effect sizes of the first two studies, the effect of the neutral text descriptions on sound pleasantness in Experiment 2 was significantly smaller than the effect of the neutral visual sources for non-misophonics in Experiment 1 (*d*_*Experiment 2*_ = 0.75, average change = 0.58; *d*_*Experiment 1*_ = 0.95, average change = 0.98; *t*(110) = -2.39, *p* = 0.019), but the effect was only marginally smaller for misophonics (*d*_*Experiment 2*_ = 0.88, average change = 1.05; *d*_*Experiment 1*_ = 1.94, average change = 1.59; *t*(43) = -1.80, *p* = 0.079).

**Fig 3:**
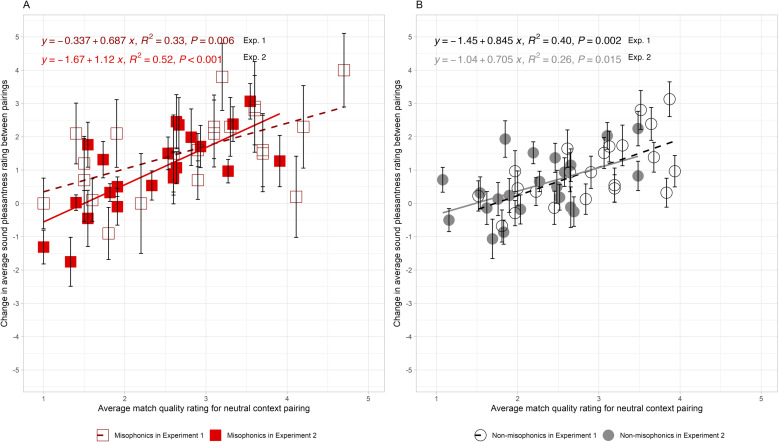
Experiments 1 and 2 quantitatively compared: Unpleasant sounds paired with neutral or unpleasant visual or text sources. These data are replotted from Figs 1 and 2. The relationship between the change in average sound pleasantness ratings across the neutral and unpleasant alternative sources for Experiment 1 movies (unfilled symbols) and Experiment 2 descriptions (filled symbols) versus the average match quality ratings for each sound-source pairing. Panel A shows both misophonic groups with squares and Panel B shows both non-misophonic groups with circles. The solid line indicates the linear regression fit to the data. Each data point represents the mean change for each of the unpleasant sounds and the error bars reflect the standard error of the mean across participants.

Given that the *change function* has a similar slope when the paired stimuli are visual sources (*β*_*Misophonics*_ = 0.69, 95% CI = [0.23, 1.15]; *β*_*Non-misophonics*_ = 0.85, 95% CI = [0.36, 1.33]) and when they are text descriptions (*β*_*Misophonics*_ = 1.12, 95% CI = [0.61, 1.62]; *β*_*Non-misophonics*_ = 0.71, 95% CI = [0.15, 1.27]), the match ratings appear to have similar meanings in both studies. This supports the interpretation that the same process of causal reassignment is happening in both studies (see Fig 3). Note, the change scores for each of the 22 sounds were marginally correlated between Experiments 1 and 2 (*r* = 0.42, R^2^ = 0.18, *F*(1, 20) = 4.30, *p* = 0.051) for misophonics, but not for non-misophonics (*r* = 0.35, R^2^ = 0.12, *F*(1, 20) = 2.72, *p* = 0.11). For misophonics, the source plausibility may be driving much of the variance, because alternative sources that have the biggest effect for movies tend to also have the biggest effect for written descriptions. This result also supports the idea that the degree of match is what determines the change in sound pleasantness. Because the match ratings are higher for the movies in Experiment 1 than for the description-sound pairs in Experiment 2 (by 0.44-points for misophonics and by 0.63-points for non-misophonics), we postulate that the visual sources increased the plausibility of the alternative source, which consequently caused a greater source reassignment. The smaller match quality in Experiment 2 would therefore explain the smaller average change in pleasantness observed in Experiment 2 than in Experiment 1.

## Experiment 3A: Altering the pleasantness of a neutral sound with neutral or unpleasant visual sources

Experiment 3A was designed to test whether the valence of the visual sources is the essential component that determines the direction of the shift in pleasantness. Given that an alternative neutral visual source can increase sound pleasantness (Experiment 1), we predicted that an alternative unpleasant visual source would decrease the pleasantness of a neutral sound. To test this idea, we paired neutral sounds from the original neutral visual sources shown in Experiment 1 with visual sources of the unpleasant events that produced the unpleasant sounds used in Experiment 1 (*N*_*s*_*U*_*v*_). We also paired the neutral sounds with their original neutral visual sources (*N*_*s*_*N*_*v*_). We predicted that neutral sounds would be rated as more pleasant when paired with their original visual sources than when paired with alternative, unpleasant visual sources. Furthermore, we predicted that better-matching unpleasant movies would be more plausible and therefore cause a greater decrease in pleasantness. However, the opposite prediction is also possible: if better-matching sound-visual pairs are more pleasant, and if more pleasant sound-visual pairs increase the pleasantness of the sound, then movies with the highest match ratings should have the highest pleasantness ratings, as seen in Experiment 1.

### Method

#### Participants.

Sixty-eight participants (M_age_ = 22.42 years; range = 18–30 years; 35 females, 31 males, two non-binary) were tested (after excluding 44 participants for failing the headphone screening). In this *unscreened* group that was recruited irrespective of misophonic status, six individuals (M_age_ = 21.83 years; range = 19–28 years; 4 females, 2 males) met our criteria for misophonia.

#### Stimuli.

The 22 neutral sounds, *N*_*s*_, combined with a video of an alternative unpleasant visual source, *U*_*v*_, to produce a movie, *N*_*s*_*U*_*v*_. This process created 22 movies (see General Methods). Additionally, the neutral sounds were combined with their original visual sources, *N*_*s*_*N*_*v*_. Our total stimulus set was 44 movies, divided equally amongst the two conditions (see Table 1).

#### Procedure.

This study followed the same procedure and design described in Experiment 1, but participants viewed *N*_*s*_*N*_*v*_ pairs and *N*_*s*_*U*_*v*_ pairs. There were 36 and 32 participants who completed the two test orders.

### Results

Experiment 3A tested the prediction that the pleasantness ratings of neutral sounds would be lower when paired with an alternative, unpleasant source (*N*_*s*_*U*_*v*_) than when paired with a visual source that depicted the original, neutral cause of the sound (*N*_*s*_*N*_*v*_). In parallel with Experiments 1 and 2, we conducted analyses only on the first half of the study so that every trial was a first exposure to a sound. In contrast to Experiment 1, given that this was an *unscreened* group, we averaged pleasantness ratings across the entire group without an analysis of misophonic status. The average sound pleasantness rating across all 22 neutral sounds in the *N*_*s*_*N*_*v*_ pairing, 0.92 (SD = 1.34) was significantly higher than in the *N*_*s*_*U*_*v*_ pairing, -1.20 (SD = 1.25) (*t*(21) = -14.60, *p* < 0.001). The average match quality rating of *N*_*s*_*U*_*v*_ pairs was 1.76 (SD = 0.63), range of 1.03 to 3.41, which was significantly lower than the average match quality rating of *N*_*s*_*N*_*v*_ pairs, 4.07 (SD = 0.67), range of 1.86 to 4.69 (*t*(21) = -12.50, *p* < 0.001). The relationship between average sound pleasantness of sound-visual pairs versus match rating is illustrated in Supplemental S8 and S9 Figs. We observe non-significant relationships for both *N*_*s*_*U*_*v*_ (R^2^ = 0.03, *F*(1, 20) = 0.62, *p* = 0.44), and *N*_*s*_*N*_*v*_ pairs (R^2^ = 0.01, *F*(1, 20) = 0.28, *p* = 0.60).

**Fig 4:**
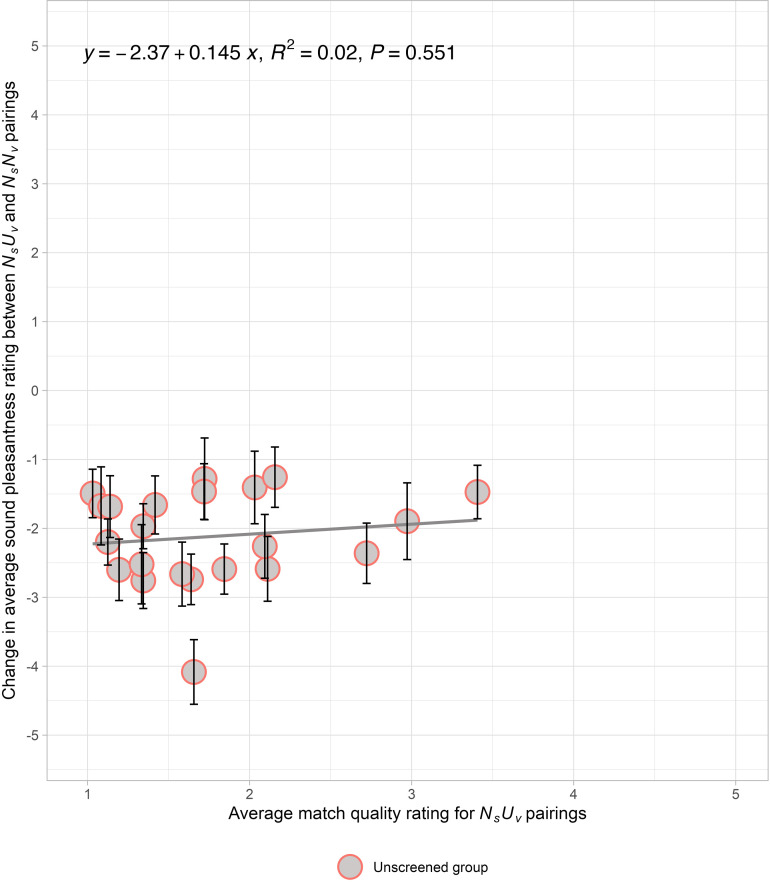
Experiment 3A: Neutral sounds paired with neutral or unpleasant visual sources. The relationship between the change in average sound pleasantness ratings across the unpleasant (*N*_*s*_*U*_*v*_) and neutral (*N*_*s*_*N*_*v*_) pairs versus the average match quality ratings for *N*_*s*_*U*_*v*_ pairs in Experiment 3A. The changes are calculated by subtracting the average pleasantness rating of *N*_*s*_*N*_*v*_ from *N*_*s*_*U*_*v*_. The averages are calculated for an *unscreened* group. The solid line indicates the linear regression fit to the data. Each data point represents the mean change for each of the unpleasant sounds and the error bars reflect the standard error of the mean across participants.

Fig 4, displaying data from the first presentation half of Experiment 3A, depicts a *change function*: the subtraction of average sound pleasantness rating of *N*_*s*_*N*_*v*_ pairs from *N*_*s*_*U*_*v*_ pairs as a function of the match quality rating of *N*_*s*_*U*_*v*_ pairs. There is no significant relationship between the change in sound pleasantness versus *N*_*s*_*U*_*v*_ match quality (R^2^ = 0.02, *F*(1, 20) = 0.37, *p* = 0.55). We observe that all 22 data points are below zero (i.e., a nearly constant negative change due to the unpleasant video). Likewise, Supplemental S10 Fig shows that the change in average sound pleasantness rating between *N*_*s*_*N*_*v*_ pairs and *N*_*s*_*U*_*v*_ pairs is not related to the average match quality rating of *N*_*s*_*N*_*v*_ pairs (R^2^ = 0.009, *F*(1, 20) = 0.18, *p* = 0.67). These comparisons show that the *N*_*s*_*U*_*v*_ pairing decreases the sound pleasantness ratings relative to the *N*_*s*_*N*_*v*_ pairing, but not as a function of match, in contrast to the significant slope relating changes in pleasantness as a function of the alternative source’s match in Experiment 1. The results in Experiment 3A are inconsistent with the *source reassignment hypothesis*, suggesting that there is another cause for the change.

We looked for evidence that the pleasantness of the visual sources were influencing the sound ratings. However, the difference in unpleasantness of the individual silent visual sources (*U*_*v*_ - *N*_*v*_) did not correlate with the change in average sound pleasantness (*N*_*s*_*U*_*v*_ - *N*_*s*_*N*_*v*_) in Experiment 3A (*r* = 0.14, R^2^ = 0.02, *F*(1, 20) = 0.42, *p* = 0.53). Therefore, there is no evidence that participants were rating visual pleasantness in instead of rating the sound pleasantness.

## Experiment 3B: Alternative meaning of auditory-visual match

Experiment 3A left open the question of how there could be a change in sound pleasantness without it having any relationship with match quality. We considered the possibility that participants were interpreting the match judgment differently between Experiment 3A and Experiment 1. In Experiment 3A, participants may have been rating the temporal alignment of the sounds and visual sources, which can be slightly misaligned due to the movie editing process. To empirically test this hypothesis, we replicated Experiment 3A with one difference: the match rating of source plausibility was followed by an evaluation of temporal match (i.e., audio and video alignment). We intended this juxtaposition of questions to isolate the factors that may have been affecting the match plausibility rating in Experiment 3A.

### Method

#### Participants.

A new set of seventeen participants were tested to replicate Experiment 3A with a modification in the procedure for match judgements (M_age_ = 19 years; range = 18–20 years; 10 females, seven males) after excluding seven participants who did not pass the headphone screening. Only one individual met our criteria for misophonia in this *unscreened* group.

#### Stimuli.

The stimuli were identical to Experiment 3A.

#### Procedure.

Experiment 3B followed the same procedure and design as in Experiment 3A, rating both sound pleasantness and match for 44 videos, but these participants made two match ratings in a row. The first match rating was a source plausibility match (worded identically to Experiment 3A), followed by a second match rating (0–4) that was a temporal match (“In this movie, are the audio and video aligned in time?”).

### Results

For this set of participants, the average change in pleasantness between *N*_*s*_*U*_*v*_ and *N*_*s*_*N*_*v*_ was 1.97 points (SD = 1.05). As found in Experiment 3A, the change in pleasantness from *N*_*s*_*U*_*v*_ to *N*_*s*_*N*_*v*_ was neither significantly related to source plausibility (R^2^ = 0.02, *F*(1, 20) = 0.45, *p* = 0.51) nor related to temporal match (R^2^ = 0.02, *F*(1, 20) = 0.41, *p* = 0.53). Next, we asked whether the plausibility and temporal match judgements were treated differently by participants. Because there was a significant correlation between plausibility and temporal match ratings (*r* = 0.63, R^2^ = 0.39, *F*(1, 20) = 12.85, *p* = 0.002), it is possible that a temporally aligned movie makes the source more plausible. Importantly, given that 61% of the variance in ratings is unique for each rating scale (because R^2^ = 0.39), these two definitions of matching are not synonymous. Overall, this experiment provides no evidence that the match rating in Experiment 3A was understood to be a temporal alignment rating, and there is no evidence that better temporal alignment caused a greater negative shift in pleasantness in Experiment 3A or 3B.

## Experiment 3C: Cross-modal agreement in Experiment 3A

Given that Experiments 3A and 3B did not find any match measure that explained the variations in sound pleasantness within conditions, Experiment 3C was designed to test whether the cross-modal agreement of the movies was causing the negative shift in sound valence. Cross-modal agreement between meaningless images and words has been found experimentally to relate to sound symbolism; for example, round visual shapes tend to match better to the word “maluma” than “takete” [39]. One possibility is that movies with better cross-modal agreement between the video and audio pairs have a greater influence on the pleasantness of the sound. To investigate this possibility, a study was conducted in which participants were instructed to categorize the sounds and video tracks into one of two categories, as a means of measuring cross-modal agreement.

### Method

#### Participants.

A total of 32 participants (M_age_ = 21.88 years; range = 18–29 years; 15 females, 13 males, four non-binary) were tested. In this *unscreened* group that was recruited irrespective of misophonic status, there were six misophonic individuals (M_age_ = 21.83 years; range = 19–28 years; 4 females, 2 males).

#### Stimuli.

There were a total of 44 silent visual tracks, and 44 sounds (22 unpleasant and 22 neutral). The two nonsense words used for cross-modal matching, “maluma” or “takete,” were chosen because they have established sound symbolism that corresponds to round or pointy shapes, respectively [39].

#### Procedure.

The experimental platform was Gorilla.sc [40]. Each sound was heard (unimodally) in random order, followed by each video source (unimodally) in random order. In subsequent data analysis, each stimulus was categorized as being either a “maluma” or a “takete” based on which name received that designation more than 50% of the time across participants. Next, every *N*_*s*_*U_*v*_* pairing used in Experiment 3A was categorized as being either in cross-modal “agreement” if the categories of the movie and the sound were the same (i.e., a “maluma” video track with a “maluma” sound, or a “takete” video track with a “takete” sound), or in cross-modal “disagreement” (i.e., a “maluma” video track was paired with a “takete” sound, or vice versa).

### Results

To test whether the cross-modal agreement in sound and visual symbolism underlies the changes in pleasantness seen in Experiment 3A, the average cross-modal match quality ratings in Experiment 3A were computed in two separate groups: one in which the sound-painting pairing agreed cross-modally, and one in which they disagreed cross-modally.

Ten (out of 22) neutral visual sources and eight (out of 22) unpleasant visual sources were categorized by more than 50% of participants as “maluma.” Inter-rater agreement was high for the neutral and unpleasant visual sources, respectively, at 0.93 (ICC Alpha, *F*(21, 611) = 16, *p* < 0.001) and 0.86 (*F*(21, 553) = 8.03, *p* < 0.001). To test whether the cross-modal agreement measured in Experiment 3C underlies the match quality ratings measured in Experiment 3A, every *N*_*s*_*U*_*v*_ pairing used in Experiment 3A was designated as being either in cross-modal “agreement” or “disagreement”, respectively, depending on ratings of each video source and the sound obtained in Experiment 3C. The average change in pleasantness of sounds that were in *N*_*s*_*U*_*v*_ pairs with cross-modal agreement (M = -2.14, SD = 0.53) was not reliably higher than the change in pleasantness for sounds that were in *N*_*s*_*U*_*v*_ pairs with cross-modal disagreement (M = -2.08, SD = 0.93) (*t*(20) = -0.22, *p* = 0.83). Therefore, cross-modal agreement based on sound symbolism does not account for significant variation in the change produced by unpleasant visual sources in Experiment 3A. Furthermore, according to a t-test for independent samples, the mean match quality rating was not significantly higher for the group of stimuli that were in cross-modal agreement (M = 1.69, SD = 0.46), than cross-modal disagreement (M = 1.88, SD = 0.89) (*t*(20) = -0.68, *p* = 0.50). This indicates that the match quality rating was not interpreted as a cross-modal agreement rating by participants.

To further test the explanatory power of cross-modal match, we conducted a parallel analysis of cross-modal effects for the neutral source movies used in Experiment 1. Because Experiment 1 had provided evidence of source reassignment, we predicted that there would be no significant correlations with cross-modal match. We found that cross-modal agreement of *U*_*s*_*N*_*v*_ pairs in Experiment 1 had no effect: the average change in pleasantness was unaffected by cross-modal agreement versus disagreement (M = 1.17, SD = 1.02 versus M = 0.85, SD = 0.85; *t*(20) = 0.52, *p* = 0.61) and was unrelated to the match (plausibility) rating (M = 2.96, SD = 0.76 versus M = 2.19, SD = 0.87; *t*(20) = 1.62, *p* = 0.12).

## Comparison of Experiments 1, 2 and 3

Experiments 1 and 2 showed that our misophonic groups rated sounds in the context of movies as more unpleasant than did non-misophonic groups. While both groups found sounds to less unpleasant when they were paired with neutral sources, this effect tended to be greater for the misophonic group (significantly in the full Experiment 1, and marginally for first exposures in Experiments 1 and 2). Although effects of the verbal descriptions were smaller than the videos, Experiments 1 and 2 were quantitatively consistent with a common mechanism of source plausibility for words and videos because they had similar *change functions*. Experiment 3A appeared to result from a different mechanism, given that its *change function* showed no relationship to source plausibility. Experiments 3B and 3C showed that the magnitude of change per video in Experiment 3A did not relate to audio-visual temporal synchrony or cross-modal agreement either, leaving open the question as to what causes the decreased pleasantness of the neutral sounds in the *N*_*s*_*U*_*v*_ movies. Taken together, Experiments 1, 2, and 3A-C constitute contrasting evidence that the neutral alternative sources caused source reassignment because variations in their pleasantness shifts correlated with match ratings based on source plausibility (but not cross-modal agreement) whereas the unpleasant visual sources did not cause source reassignment because variations in their pleasantness shifts did not correlate with match ratings.

## Experiment 4: The role of sound misidentification in the effectiveness of neutral visual sources

We considered whether participants in Experiments 3A-C rate the pleasantness of the unpleasant visual sources (rather than the sounds); if so, this predicts that there should be a high correlation between *U*_*v*_ video and *N*_*s*_*U*_*v*_ sound pleasantness. This question motivates an experiment to measure the pleasantness and identification of *U*_*s*_ and *N*_*s*_ (Experiment 4) and the pleasantness of *U*_*v*_ and *N*_*v*_ (see Supplementary File S2). Experiment 4 serves two purposes. First, it asks whether inherent sound ambiguity might permit an alternative visual source to be more plausible and effective for that sound. Sound ambiguity was tested in a sound identification experiment that measured the rate at which each sound was misidentified as its alternative source. Cases of misidentification allow us to test a prediction that is made exclusively by the source-reassignment explanation and not by a visual pleasantness explanation: the ambiguous unpleasant sounds which tend to be misheard as a neutral sound should be rated as matching well to an alternative neutral visual source, while the ambiguous neutral sounds should be rated as matching well to an alternative unpleasant visual source. Second, the study design contains two measurements of sound-alone pleasantness, allowing us to test whether the sounds’ pleasantness changes upon second listening. Although the “mere exposure” effect is well-known for increasing stimulus preference, there is some evidence (e.g., Brickman et al. [41]) showing that the mere exposure effect applies differentially to positive/neutral stimuli and negative stimuli, with mildly negative stimuli becoming more negative upon repeated exposure. Experiment 4 measures both the identifiability and the “mere exposure” effect for all the neutral and unpleasant sounds used in our studies.

### Method

#### Participants.

Thirty-two participants (M_age_ = 24.40 years; range = 18–30 years; 12 females, 20 males) were tested (after excluding 13 participants for failing headphone screening). In this *unscreened* group, 11 participants (M_age_ = 25.18 years; range = 18–29 years; five females, six males) met the criteria for misophonia.

#### Stimuli.

There were 22 unpleasant sounds, *U*_*s*_ and 22 neutral sounds, *N*_*s*_ (See Table 1 and General Methods). Each of the 44 sounds were presented in isolation.

#### Procedure.

During the first block of 44 trials, participants were asked to rate the pleasantness of one sound per trial (i.e., a first exposure). The same pleasantness scale as Experiment 1 was used. In the subsequent block of 44 trials, participants rated the pleasantness of each sound again (i.e., a second exposure) before identifying it by selecting one label from a closed set of 10 labels [42]. The labels consisted of a noun and a verb taken from a descriptive phrase (see Table 1). The 10 labels were randomly selected on each trial from the entire set of 44 possible labels, with the restriction that two of the ten labels were always (1) the correct answer and (2) the corresponding alternative sound. The presentation order of the sounds was random.

### Results

Experiment 4 tested the identification of our unpleasant and neutral sounds and tested the effects of repetition on pleasantness. We first apply this data to Experiment 1. Sound identification accuracy of *U*_*s*_ was 77.0% (SD = 0.20) with a range from 40.6% for *person cracking their knuckles* to 100% for *person wheezing* and *person scraping a knife and fork together*. Table 2 shows the frequency of misidentifications for each unpleasant sound. In most instances of misidentification, unpleasant sounds were misidentified as their planned neutral counterparts (Planned source, 3^rd^ column from right). The odds ratio is 3.09 for planned sources versus 0.05 for unplanned sources, which was calculated by dividing the number of misidentifications per type (planned or other source) by the number of total participants in the study. These instances should, in principle, raise the average *U*_*s*_ pleasantness ratings. This leads to the prediction that the rate at which *U*_*s*_ are confused for their planned neutral counterparts should correlate with higher average *U*_*s*_ pleasantness ratings. This prediction was upheld by a significant correlation (*r* = 0.20, *F*(1, 20) = 5.012, *p* = 0.036, first exposure). In effect, this result means that the true unpleasantness of ambiguous *U*_*s*_ sounds are underestimated in our sound-alone condition relative to a situation in which the source is known or strongly implied (i.e., as in Experiments 1 and 2 via visual or text input). Furthermore, we reasoned that a sound which is *sometimes* spontaneously confused for its planned neutral counterpart should *often* be considered plausible when it is paired with that visual source. This reasoning predicts the significant correlation we found between rate of confusion of each *U*_*s*_ and its average match rating within the *U*_*s*_*N*_*v*_ pairing from all participants in Experiment 1 (*r* = 0.61, *F*(1, 20) = 12.30, *p* = 0.002, first exposure) as well as its change in pleasantness between the *U*_*s*_*N*_*v*_ and *U*_*s*_*U*_*v*_ conditions (*r* = 0.56, *F*(1, 20) = 9.36, *p* = 0.006, first exposure).

**Table 2:** Average identification accuracy and average misidentification rate for each unpleasant sound across all participants (*unscreened* group).

			Misidentification Instances		
			*Neutral sources*		*Unpleasant source*
**Sound Name**	**Identification Accuracy (%)**	**Misidentification**^a^ **Rate (%)**	**Planned source**^b^ **[1]**	**Other source**^c^ **[43]**	**Other source** ^d^
Person smacking their lips	84.4	15.6	1	3	1
Person brushing their teeth	46.9	53.1	15	2	0
Person eating chips	62.5	37.5	4	6	2
Person crinkling a plastic bottle	56.3	43.8	11	0	3
Person cracking their knuckles	40.6	59.4	17	1	1
Person sniffing 1	78.1	21.9	3	3	1
Person scraping a fork and knife together	100.0	0.0	0	0	0
Person sniffing 2	59.4	40.6	6	5	2
Person typing on a keyboard	93.8	6.3	2	0	0
Person sucking in air through their teeth	78.1	21.9	2	2	3
Person coughing	100.0	0.0	0	0	0
Person chewing gum	93.8	6.3	1	0	1
Person swishing water in their mouth	81.3	18.8	4	1	1
Person scratching scalp (far away)	81.3	18.8	2	3	1
Person gulping water	96.9	3.1	0	0	1
Person wheezing	100.0	0.0	0	0	0
Person sniffing (noisily breathing)	75.0	25.0	7	1	0
Person sneezing	90.6	9.4	0	1	2
Person scratching a blackboard	50.0	50.0	13	3	0
Person blowing their nose	59.4	40.6	4	3	6
Person sipping through a straw	84.4	15.6	3	0	2
Person tapping fingers on table	87.5	12.5	4	0	0
**Total number of misidentifications**	**544**		**99**	**34**	**27**
**Odds Ratio** ^**e**^	**17**		**3.09**	**0.05** ^**f**^	

**See** S3 Table **for identification accuracy of neutral sounds.**

^a^Misidentification refers to confusing the sound for its planned neutral counterpart or for any other sound.

^b^Number of participants who misidentified the unpleasant sound for its planned, neutral counterpart.

^c^Number of participants who misidentified the unpleasant sound for a neutral source that was not the planned counterpart.

^d^Number of participants who misidentified the unpleasant sound for another unpleasant sound.

^e^Odds ratio is calculated by dividing the number of misidentifications per type (e.g., planned neutral source) by the number of total participants in the study.

^f^Odds ratio for the ‘other source’ category is calculated over a combined pool of the neutral and unpleasant instances.

Second, we apply this data to Experiment 3A. Sound identification accuracy of the *N*_*s*_ was 82.0% (SD = 0.20) with a range from 44.0% for sound of a *person snapping a stick* to 100% for sounds of *campfire burning, birds chirping*, person pulling facial *tissues out of a box*, *person tapping a bag that is laying on top of a tambourine*, and *stream flowing*. S3 Table shows that the odds ratio is 1.00 for planned sources versus 0.07 for unplanned sources. These confusions of planned neutral sounds with unplanned unpleasant sources should, in principle, lower the average *N*_*s*_ pleasantness ratings. This leads to the prediction that the rate at which the *N*_*s*_ are confused for their planned unpleasant counterparts should correlate with *lower* average *N*_*s*_ pleasantness ratings. However, this prediction was not upheld (*r* = 0.15, *F*(1, 20) = 0.472, *p* = 0.50, first exposure). There was no correlation between rate of confusion of each *N*_*s*_ with its average match rating within the *N*_*s*_*U*_*v*_ pairing from Experiment 3A (*r* = 0.29, *F*(1, 20) = 1.83, *p* = 0.19, first exposure), nor with its change in pleasantness between the *N*_*s*_*U*_*v*_ and *N*_*s*_*N*_*v*_ conditions (*r* = 0.005, *F*(1, 20) = 0.0006, *p* = 0.98, first exposure).

The identification and pleasantness data permit us to quantitatively estimate how much a sound source reassignment could change the pleasantness of the sound in Experiment 1. Because the average pleasantness of correctly identified unpleasant sounds during first exposure was -1.44 (SD = 1.58), and the average pleasantness of their correctly identified neutral counterpart sounds was 0.28 (SD = 1.34), we estimated that the largest possible change in pleasantness caused purely by source reassignment would be their difference, 1.72 points. This difference provides an upper bound on the size of the effect that could be obtained in Experiment 1, assuming all *U*_*s*_ sounds were correctly identified in the *U*_*s*_*U*_*v*_ trials and fully reassigned to neutral sound sources when accompanied by neutral movies. This upper bound of the effect is large enough to account for the shifts obtained in Experiment 1 because the average change in sound pleasantness was 1.13 points (subtracting the *U*_*s*_*N*_*v*_ of -0.41 from the *U*_*s*_*U*_*v*_ of -1.54, first exposure across all participants). This rules out the need to appeal to any additional mechanism aside from source reassignment to account for the size of the changes in pleasantness that were observed in Experiment 1.

To examine how repeated exposure affects the pleasantness these sounds, we conducted a repeated measures ANOVA to compare sound pleasantness ratings across sound valence (*U*_*s*_ or *N*_*s*_) and exposure (first or second). The average pleasantness rating for each sound was calculated by averaging the rating across all participants, irrespective of whether the sound was correctly identified. This calculation was completed separately for each sound valence and exposure. The mean pleasantness of *U*_*s*_ (M_first_ = -1.34, SD_first_ = 1.51; M_second_ = -1.48, SD_second_ = 1.58) was significantly lower than the pleasantness of *N*_*s*_ (M_first_ = 0.24, SD_first_ = 1.32, M_second_ = 0.48, SD_second_ = 1.41) (*F*(1, 42) = 16.69, *p* < 0.001, *η*_*p*_^*2*^ = 0.284, power = 0.980). We did not observe a main effect of first versus second exposure (*F*(1, 42) = 0.787, *p* = 0.38, *η*_*p*_^*2*^ = 0.018, power = 0.140). More importantly, we did observe a significant interaction between sound valence and exposure (*F*(1, 42) = 10.50, *p* = 0.002, *η*_*p*_^*2*^ = 0.200, power = 0.886). Pleasantness ratings for *U*_*s*_ were 0.14 points lower during second exposure, whereas the pleasantness ratings for *N*_*s*_ were 0.25 points higher during second exposure. This result agrees with our prediction that the mere exposure effect applies differentially to positive/neutral stimuli and negative stimuli, with mildly negative stimuli becoming more negative upon repeated exposure.

## Experiment 5A: Altering the pleasantness ratings of sounds with meaningless visual stimuli

Experiment 1 provided evidence that an unpleasant sound is rated as more pleasant when paired with an alternative neutral visual source than when it is presented with an unpleasant visual source. *The source reassignment hypothesis* is that the visual source changes the perceived cause of the sound, and this explains why the change is greater when there is a better match between the visual source and the sound. However, the alternative visual sources may also have the potential to change the ratings of the sounds by other mechanisms, such as contaminating the sound ratings with their visual pleasantness. Although the results of Experiment 1 did not show an effect of the pleasantness of the silent visual source on the change function, those visual sources had the same source as the sounds and therefore shared meaning. Because the meaning of the sound source is a strong factor in its emotional effect, it is possible that the visual source’s semantics (meaning of the source) overwhelmed the effect of visual pleasantness Therefore, Experiment 5A was devised to directly test the potential alternative mechanism of perceptual visual pleasantness devoid of meaning. In this study, we paired pleasant abstract paintings with our unpleasant sounds because they contained no semantic content. Furthermore, because these were static images, there was no auditory-visual temporal asynchrony introduced by showing unrelated visual input.

### Method

#### Participants.

Twenty participants (M_age_ = 19.76 years; range = 18–22 years; 17 females, two males, one non-binary) were tested irrespective of misophonic status (after excluding five participants for failing the headphone screening). In this *unscreened* group, four participants (M_age_ = 20 years; range = 18–22 years; 3 females, 1 male) met the criteria for misophonia.

#### Stimuli.

The 22 *U*_*s*_ sounds from Experiment 1 were played simultaneously with pleasant, abstract paintings, *U*_*s*_*P*_*p*_ (See General Methods and Table 1). Between participants, the pairing of unpleasant sounds to the abstract paintings was random so that each listener experienced a custom set of *U*_*s*_*P*_*p*_ pairs. We used 166 abstract paintings from The Art Institute of Chicago online collection [43] and Pexels [44], a free stock photo website.

#### Procedure.

It has been shown that the perception of abstract art differs substantially across individuals [45], i.e., there is no consensus on whether an abstract piece of art is pleasant or unpleasant. Therefore, this experiment was preceded by a pretest (Part One) to select paintings that would be pleasant for each participant. In Part One, each participant rated the pleasantness of 166 abstract paintings. Each painting was viewed for 12 seconds, the average duration of the unpleasant sounds, before being given a pleasantness rating on the same 11-point scale described in Experiment 1. For each participant, the 22 abstract paintings with the most positive pleasantness ratings were selected. The preselected paintings and sounds were randomly paired and displayed throughout the duration of the sound using iMovie [46]. Approximately four days later, in Part Two, the participants completed ratings of the sounds with and without accompanying images. They first rated the pleasantness of all the unpleasant sounds in isolation, using the 11-point pleasantness scale; next, they observed the *U*_*s*_*P*_*p*_ pairs and rated the sound pleasantness as well as the match quality of *U*_*s*_*P*_*p*_ pairs. Instructions were to rate “How well the sound matches the painting.” The presentation order of the stimuli within their respective sections was random. In an additional step after the experiment, 11 of the participants again rated the pleasantness of the silent paintings. All other procedural elements (i.e., survey via Qualtrics, volume calibration, headphone screening, and catch trials) were the same as in the common procedures in General Methods.

### Results

**Fig 5:**
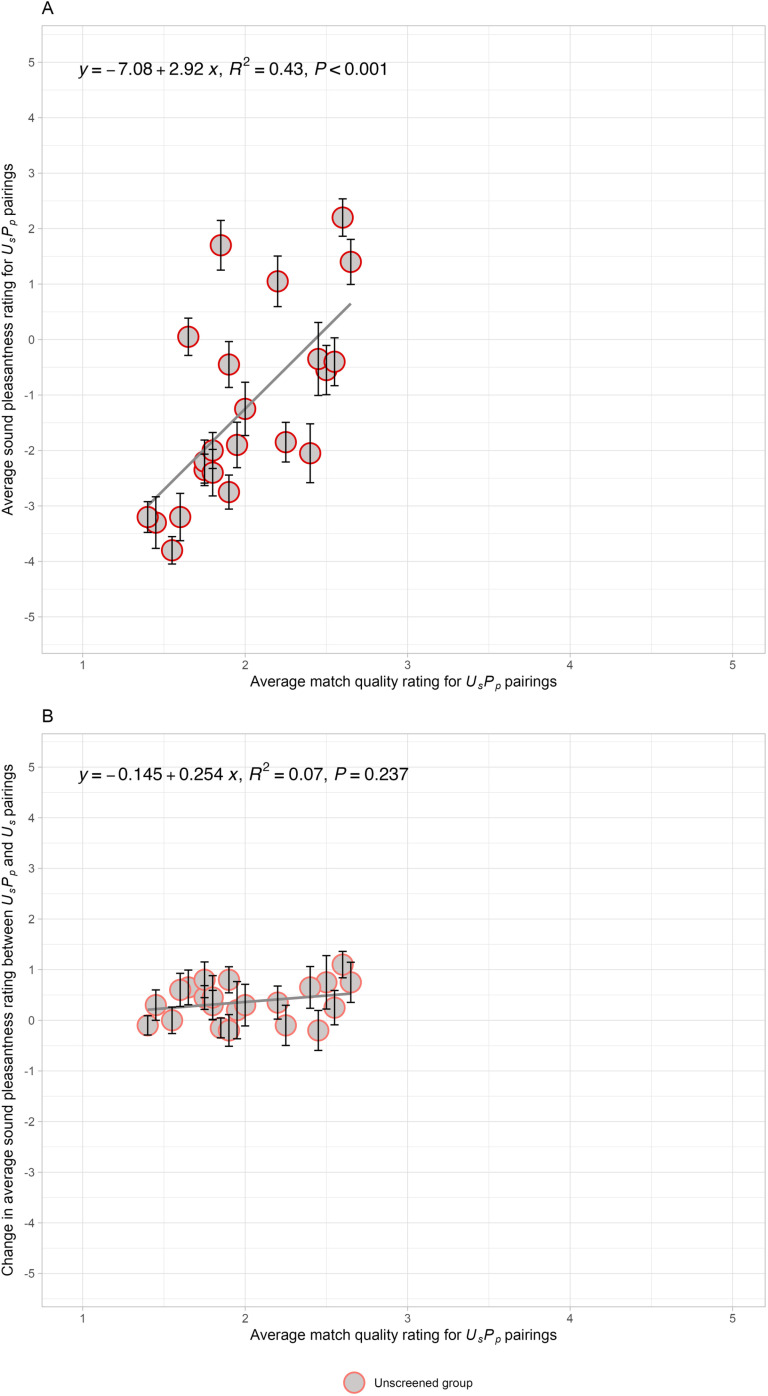
Experiment 5A: Unpleasant sounds paired with neutral or unpleasant visual sources. (A) The relationship between average sound pleasantness ratings for *U*_*s*_*P*_*p*_ pairs versus average match quality ratings for *U*_*s*_*P*_*p*_ pairs in Experiment 5A. The solid line indicates the linear regression fit to the data. Each data point represents the mean rating across observers for one unpleasant sound, while the error bar reflects the standard error of the mean. (B) The relationship between the change in average sound pleasantness ratings across the two pairs (*U*_*s*_*P*_*p*_ or *U*_*s*_) versus average match quality ratings for *U*_*s*_*P*_*p*_ pairs in Experiment 5A. The changes are calculated by subtracting the average pleasantness rating of *U*_*s*_ from *U*_*s*_*P*_*p*_. The solid line indicates the linear regression fit to the data. The 22 data points represent the mean change for each of the unpleasant sounds and the error bars reflect the standard error of the mean across participants.

Experiment 5A tested the alternative hypothesis that the pleasantness of an unpleasant sound, *U*_*s*_, would increase when it was presented simultaneously with a pleasant but semantically unrelated painting, *P*_*p*_. The average pleasantness rating for each *U*_*s*_*P*_*p*_ across the entire *unscreened* group was calculated by averaging the sound pleasantness ratings irrespective of the painting with which the sound was paired. The mean pleasantness of the sound alone (M = -1.62, SD = 1.72) was significantly lower than the pleasantness of the sound in the *U*_*s*_*P*_*p*_ pairing (M = -1.25, SD = 1.73; *t*(21) = -4.51, *p* < 0.001). Fig 5A shows that the average sound *pleasantness* ratings in the *U*_*s*_*P*_*p*_ pairing did increase as a function of the average match quality of *U*_*s*_*P*_*p*_ pairs (R^2^ = 0.43, *F*(1, 20) = 15.10, *p* < 0.001, slope of the function = 2.92). The average match quality rating was 2.00 (SD = 0.39) with a range from 1.40 to 2.65. Next, to illustrate the relationship between pairing and match, Fig 5B depicts a *change function*: the subtraction of the sound pleasantness rating when in isolation from the sound pleasantness rating when in *U*_*s*_*P*_*p*_ pairing as a function of the match quality ratings of *U*_*s*_*P*_*p*_ pairs. The *change function* has a non-significant horizontal line-of-best-fit, indicating that a change in sound pleasantness is not associated with greater match quality (R^2^ = 0.069, *F*(1, 20) = 1.49, *p* = 0.24, slope of the function = 0.25).

Additionally, the pleasantness of the abstract paintings decreased significantly after viewing them with the unpleasant sound (for the 11 participants who completed that condition) (M_FIRST_ = 3.79, SD = 0.23; M_SECOND_ = 1.81, SD = 0.73) (paired-sample *t*(21) = 14.33, *p* < 0.001).

The pleasantness of abstract paintings (measured in Part One) did not account for variance in judgements of sound pleasantness of *U*_*s*_*P*_*p*_ pairs (R^2^ = 0.015, *F*(1, 20) = 0.29, *p* = 0.59), nor match quality (R^2^ = 0.01, *F*(1, 20) = 0.24, *p* = 0.63), nor did it correlate significantly with pleasantness ratings when the sound was presented alone (R^2^ = 0.002, *F*(1, 20) = 0.035, *p* = 0.85). In Part One, the pleasantness of the paintings, across all 20 participants, had a mean of 3.46 (SD = 0.19) with a range of 3.10 to 3.85, which may have increased the sound pleasantness by as much as 0.40 points.

## Experiment 5B: Altering the pleasantness of an unpleasant sound with concurrent presentation of a neutral visual source

In Experiment 5B, we predicted that our original set of movies depicting alternative neutral visual sources (*U*_*s*_*N*_*v*_) could increase the perceived pleasantness of our unpleasant sounds relative to the sounds alone. Experiment 5B uses the same procedure as Part Two in Experiment 5A to permit a quantitative comparison of effect sizes between the two studies while also serving as a replication of our findings in Experiment 1. The study design of Experiments 1–3 limits our ability to measure the direct effect of the neutral visual source because the sounds are always played with a video. The present study design allows us to test whether the entire relative effect in Experiment 1, obtained by subtracting two conditions containing different video tracks, is due to only one type of visual source (e.g., unpleasant videos).

### Method

#### Participants.

Thirty-four participants (Mage = 19.70 years; range = 18–31 years; 23 females, 11 males) participated irrespective of misophonia status (after excluding 9 participants for failing the headphone screening). In this *unscreened* group, there were five misophonics (M_age_ = 19.40 years; range = 18–20 years; 4 females, 1 male).

#### Stimuli.

There were 22 unpleasant sounds, *U*_*s*_, and 22 *U*_*s*_*N*_*v*_ movies (see Table 1).

#### Procedure.

The procedure was identical to Part Two of Experiment 5A except that neutral movies were paired with sounds instead of paintings. In the first half of the study, participants rated the sounds alone. In the second half of the study, participants observed *U*_*s*_*N*_*v*_ movies and rated both the pleasantness of the sound and the match quality of the movie. The presentation order of the stimuli within their respective sections was random.

### Results

**Fig 6:**
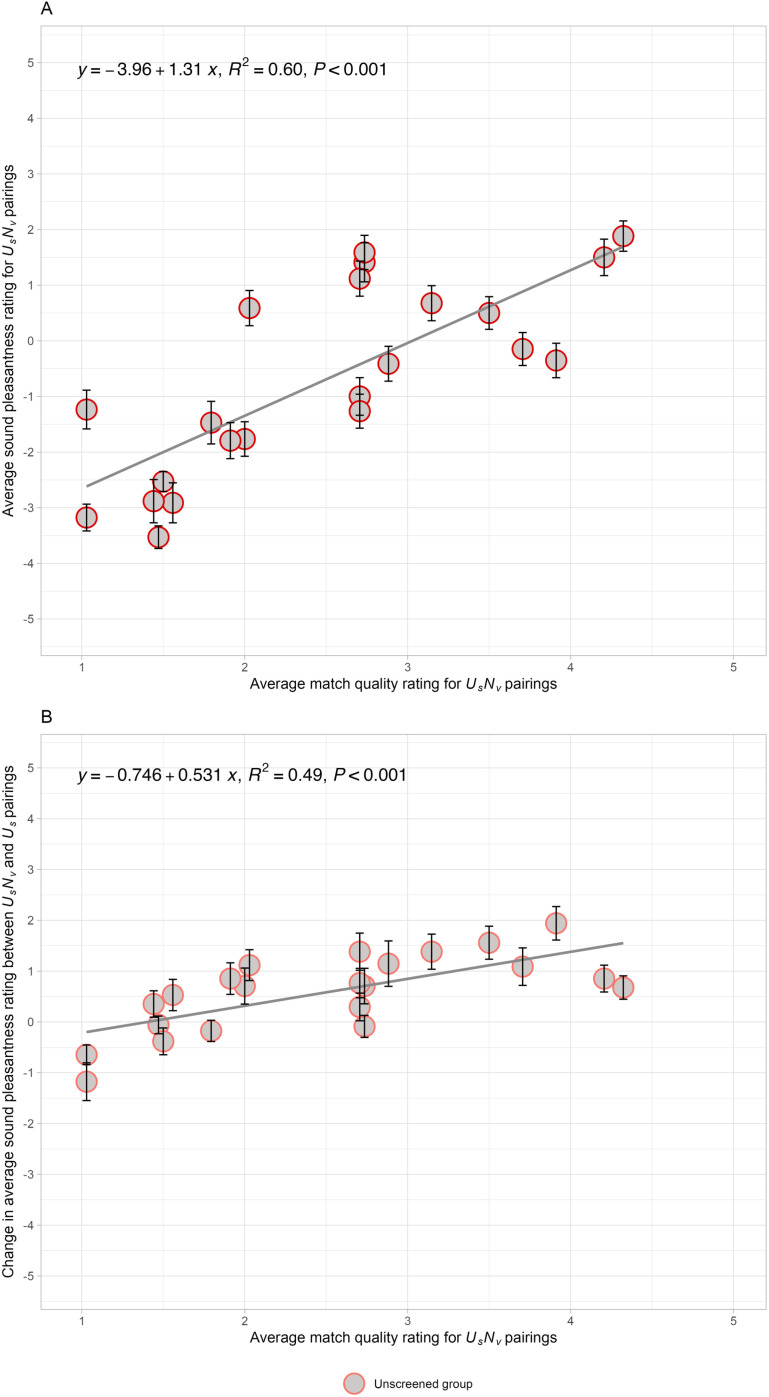
Experiment 5B: Unpleasant sounds alone and paired with neutral visual sources. (A) The relationship between average sound pleasantness ratings for *U*_*s*_*N*_*v*_ pairs versus average match quality ratings for *U*_*s*_*N*_*v*_ pairs in Experiment 5B. The solid line indicates the linear regression fit to the data. Each data point represents the mean rating across observers for one unpleasant sound, while the error bar reflects the standard error of the mean. (B) The relationship between the change in average sound pleasantness ratings across the two pairs (*U*_*s*_*N*_*v*_ or *U*_*s*_) versus average match quality ratings for *U*_*s*_*N*_*v*_ pairs in Experiment 5B. The changes are calculated by subtracting the average pleasantness rating of *U*_*s*_ from *U*_*s*_*N*_*v*_. The solid line indicates the linear regression fit to the data. The 22 data points represent the mean change for each of the unpleasant sounds and the error bars reflect the standard error of the mean.

Experiment 5B tested the prediction that pleasantness of a sound in isolation (*U*_*s*_) would increase when it was paired with a visual source that offered a neutral causal explanation of the sound (*U*_*s*_*N*_*v*_). Averaged across the *unscreened* group, the mean pleasantness of the sounds alone (M = -1.27) was significantly lower than the pleasantness of the sound in the *U*_*s*_*N*_*v*_ pairing (M = -0.69) (*t*(21) = -3.58, *p* = 0.001). Fig 6A shows the average sound pleasantness ratings in the *U*_*s*_*N*_*v*_ pairing as a function of the average match quality of *U*_*s*_*N*_*v*_ pairs (R^2^ = 0.60, *F*(1, 20) = 29.63, *p* < 0.001). In agreement with previous findings, the average sound pleasantness in the *U*_*s*_*N*_*v*_ pairs increased with greater match quality ratings. The average match quality rating was 2.50 (SD = 1.00) with a range from 1.03 to 4.32. The means for all stimuli are available in Supplementary File S2. Lastly, to illustrate the relationship between the effectiveness of a *U*_*s*_*N*_*v*_ pairing and its match, Fig 6B depicts a *change function*: the pleasantness rating of *U*_*s*_ subtracted from the sound pleasantness rating of *U*_*s*_*N*_*v*_ as a function of the match quality rating of *U*_*s*_*N*_*v*_ pairs. The best-fitting line to the data shows that a greater match quality is associated with a greater effect of the neutral visual source (R^2^ = 0.49, *F*(1, 20) = 19.03, *p* = 0.003). At the lowest match quality rating (1), the change in sound pleasantness is approximately -0.21. Thereafter, the change in sound pleasantness increases by 0.53 points with every 1-point increase in match quality rating. We note that the sounds with the largest pleasantness change for our *unscreened* group were: *a person smacking their lips, a person eating chips, a person cracking their knuckles,* and *a person sniffing 2*, which changed by 1.94, 1.56, 1.38, and 1.38 points, respectively.

Next, the results of Experiment 5B were harnessed to assess the reliability and validity of our pleasantness ratings across studies. Ratings of sounds isolation (*U*_*s*_) were highly correlated between Experiments 5B and 4 (first exposure) (*r* = 0.94, *F*(1, 20) = 164.34, *p* < 0.001), and between Experiments 5B and 5A (*r* = 0.97, *F*(1, 20) = 311.72, *p* < 0.001). We also found a significant correlation between sound pleasantness ratings of *U*_*s*_*N*_*v*_ pairings in Experiment 5B and 1 (across all participants, first exposure, *r* = 0.93, *F*(1, 20) = 123.40, *p* < 0.001), as well as for match quality ratings of *U*_*s*_*N*_*v*_ pairings in Experiment 5B and 1 (*r* = 0.94, *F*(1, 20) = 145.88, *p* < 0.001). We attribute the high reproducibility of our data to our strict headphone screening process and our catch trials. The convergent validity of the unimodal ratings of each sound source was shown by the significant correlation between *U*_*s*_ pleasantness ratings from Experiment 5B and the baseline *U*_*v*_ pleasantness ratings (*r =* 0.70*, F*(1, 20) = 19.66*, p* < 0.001, Supplemental File S2). Furthermore, conditions in study 5B provided the data needed to test whether the duration of our sounds had any effect on pleasantness (see Supplemental File S2). Duration did not correlate with sound pleasantness in either the *U*_*s*_ or *U*_*s*_*N*_*v*_ conditions.

## Comparison of Experiments 5A and 5B

The abstract pleasant paintings in Experiment 5A caused a small, yet reliable, increase in pleasantness of the sounds. This could support the hypothesis that visual pleasantness influences sound ratings. Importantly, although the match quality of *U*_*s*_*P*_*p*_ is correlated to the sound pleasantness of *U*_*s*_*P*_*p*_, its match quality does not relate to the *change* in pleasantness, *U*_*s*_*P*_*p*_ *- U*_*s*_. The size of the change in sound pleasantness produced by the paintings (0.37 points), although reliable, is smaller than the change produced by video sources in Experiment 5B (0.58 points), even though the paintings are more visually pleasant (3.46 points) than the videos (1.29 points in Supplemental File S2). This comparison shows an advantage in the potency of source reassignment over visual pleasantness. As an aside, we note that the pleasantness of the paintings decreased in their second silent exposure, potentially indicating that the intervening *U*_*s*_*P*_*p*_ condition may have formed associations between the paintings and the unpleasant sounds. In Experiment B it was expected that there would be a significant correlation between video only and sound only pleasantness, given their common meanings in terms of source events; however, we note that with *r* = 0.70, half of the variance amongst stimuli is specific to the perceptual modality and individual stimulus properties rather than being entirely determined by their common source events.

Both Experiments 5A and 5B expose listeners to the same sounds twice. This leaves open the possibility that ratings could increase with visual stimuli purely due to repeated exposure. However, because Experiment 4 found that our unpleasant sounds did not become more pleasant upon second exposure, we conclude that the results of Experiments 5A and 5B reflect true increases in sound pleasantness due to the visual stimuli in the second half.

There is a substantial difference between Experiments 5A and 5B (cf. Figs 5B and 6B). Plotting the relationship between *change* in sound pleasantness and match quality removes effects of the sound’s inherent pleasantness. It shows that the mere correlation between match and pleasantness in these experiments (cf. Figs 5A and 6A) does not provide evidence that a better match *causes* a stronger source reassignment. We posit that a strong relationship between *change* in sound pleasantness and match quality implies that the visual stimulus is reassigning the source. Therefore, a source reassignment hypothesis is supported for videos (Fig 6) whereas it is not supported it for paintings (Fig 5). This result left open the question of what caused the change in sound pleasantness for paintings.

## Experiment 5C: Cross-modal agreement and match ratings

The results of Experiment 5A led to further questions regarding the meaning of the match ratings. Firstly, why were many of the sound-painting match ratings greater than 1 (on a scale of 1–5) for randomly paired, meaningless visual stimuli? Experiment 5C tested whether the match ratings in Experiment 5A indicate the degree of cross-modal agreement between the painting and the sound. Secondly, why do the match ratings correlate *at all* with the pleasantness ratings of the sounds within the painting-sound pairs? One possibility is that paintings with better cross-modal agreement in the *U*_*s*_*P*_*p*_ pairs have a greater influence on the pleasantness of the sound. To investigate these questions, a study analogous to the cross-modal study in Experiment 3C was conducted. Participants were instructed to categorize each of the sounds and paintings as matching the word “maluma” or “takete” as a means of measuring cross-modal agreement.

### Method

As part of the study reported in Experiment 3C, the same 32 participants (M_age_ = 21.88 years; range = 18–29 years; 15 females, 13 males, four non-binary) judged whether the name “maluma” or “takete” best matched the 144 paintings used in Experiment 5A. See Experiment 3C Methods for further details. After each stimulus was categorized as being either a “maluma” or a “takete,” every *U*_*s*_*P*_*p*_ pairing was categorized as being either in cross-modal “agreement” (e.g., “maluma sound with “maluma” painting) or in cross-modal “disagreement” (e.g., “maluma” sound with a “takete” painting).

### Results

Experiment 5C tested the prediction that the increase in *U*_*s*_ pleasantness resulting from abstract paintings would relate to their individual cross-modal agreement in sound symbolism. Seven unpleasant sounds, eight neutral sounds, and 77 pleasant paintings were rated by more than 50% of the participants as “Maluma.” Inter-rater agreement (ICC Alpha) for unpleasant sounds, neutral sounds, and paintings, respectively, was 0.85 (*F*(21, 650) = 7.07, *p* < 0.001), 0.92 (*F*(21, 675) = 12.50, *p* < 0.001) and 0.88 (*F*(143, 2829) = 8.82, *p* < 0.001). According to a t-test for independent samples, the mean match quality rating was significantly higher for the sounds and paintings that were in cross-modal agreement (M = 2.28, SD = 1.23), than cross-modal disagreement (M = 1.74, SD = 0.90), (*t*(366) = 4.87, *p* < 0.001). The mean change in pleasantness for sounds that were in pairs with cross-modal agreement (M = 0.56, SD = 1.49) was also reliably higher than the change in pleasantness for sounds that were in pairs with cross-modal disagreement (M = 0.21, SD = 1.65), (*t*(366) = 2.07, *p* = 0.039). Therefore, *cross-modal agreement* based on sound symbolism *does account for significant variation* in the match ratings in Experiment 5A, which offers an explanation as to why there were modest match ratings in that study. Additionally, because cross-modal agreement did predict the size of the effect from a pleasant painting, it also offers a mechanism for how paintings increased sound pleasantness without reassigning the sound source.

As stated earlier, the *source reassignment hypothesis* posits that the neutral visual sources cause the observer to reassign the source of the sound to the depicted event, thereby increasing the perceived pleasantness of the sound. Experiments 1 and 5B revealed that alternative neutral visual sources can change the pleasantness of the sound as a function of how well the neutral visual source and sound match. We did not see such a *change function* in Experiment 5A; sound pleasantness increased or decreased regardless of the match of an abstract painting. The significant slope relating changes in pleasantness as a function of match in Experiment 5B is consistent with the *source reassignment hypothesis*, whereas changes in intercept only, seen in Experiment 5A, are inconsistent with it.

## General discussion

Altogether, these experiments indicate that the perceived pleasantness of a sound can be modulated by pairing the sound with visual or semantic input, but the mechanisms for this change differ between conditions. A shift in sound pleasantness can be achieved by: (1) combining a sound with a dynamic visual source, (2) combining a sound with a text description of an alternative source, and (3) combining an unpleasant sound with a pleasant but meaningless visual image. To support these and future studies, we describe and validate a new database of openly available stimuli.

Our finding that neutral videos increase the pleasantness of sounds agrees with past research [12,16,23]. We found that the increase caused by neutral videos was significantly greater for a misophonic group when we looked across our entire study, which differs from [16] which found differences in bodily sensations but not a greater effect on pleasantness for their misophonic group. Testing order is a possible reason for the difference, because they used a within-participants design in which all the positive videos were seen first, whereas our design showed half the positive and half the negative videos first. As found by others [23], we found that the response to a video depended on what had preceded it. Although the effect of neutral text descriptions on the pleasantness of an unpleasant sound is smaller than the effect of videos of neutral visual sources, text descriptions do produce robust effects that are consistent with source reassignment. Our results agree with past studies using words [10,11]. An advantage of our studies is that we show that both our visual sources and descriptions exhibit the same quantitative relationship, suggesting that they are subserved by the same mechanism. Furthermore, we show that text descriptions work for misophonic participants as well as for non-misophonics. While our sounds are mostly well-identified in isolation, ambiguity tends to help an unpleasant sound to match with, and be affected by, an alternative source. This result agrees with others showing the importance of trigger identification [6–8]. A strength of our studies is that they show that the quantitative difference between the pleasantness of the unpleasant sounds and their neutral counterparts is large enough to account for the size of the pleasantness shift caused by visual sources.

Furthermore, although sound pleasantness can be altered by meaningless pleasant visual input, we conclude that visual pleasantness is not the primary underlying mechanism for the beneficial effect of the neutral visual sources. This is because visual pleasantness and source reassignment have different effects on the *change function*. If the visual/semantic input alters the perceived source of the sound, the magnitude of the change in sound pleasantness associated with changing the visual/semantic input should vary systematically as a function of the plausibility match between a sound and its visual/semantic input. While other studies have measured audio-visual match [12,16,23], we propose a novel way to confirm source reassignment with a *change function*. We observe a *change function* when neutral visual sources and descriptions are paired with unpleasant sounds (as in Experiments 1, 2, and 5B). In contrast, if visual input does not alter the perceived source of the sound, the plausibility match ratings should be unrelated to the magnitude of the change, even if the overall mean does shift. We observe this alternative pattern when pleasant unrelated images are paired with unpleasant sounds (Experiment 5A) and when unpleasant visual sources are paired with neutral sounds (Experiment 3A).

Because the relationship between source plausibility and change in pleasantness is central evidence for source reassignment, it is important to empirically test the meaning of the plausibility match rating when source reassignment is not evident. First, we asked why there could be a match without a meaningful visual stimulus in Experiment 5A. We found evidence that the weak-moderate match ratings between the abstract paintings and sounds were attributable to cross-modal agreement. Second, we showed with Experiments 3B and 3C that the match rating based on source plausibility in Experiment 3A was not simply a rating of audio-visual temporal alignment, nor was it a reflection of cross-modal agreement. Overall, conditions that show evidence of source reassignment do not show the effects of cross-modal agreement, and vice-versa.

Finally, we ask whether the pleasantness ratings reflect feelings about the sounds, or the sources of the sounds. The high agreement in the change versus match function for Experiments 1 and 2 suggest that, for a given level of match, the source reassignment process produced the same pleasantness regardless of whether visual stimuli or written descriptions depicted the event.

One limitation of this series of studies is that we included all stimuli in all studies, even after our first study indicated which stimuli were most effective. We did this for hypothesis testing, which required a wide range of movie matches and effectiveness. However, this approach is not ideal for applications which aim to maximize effect sizes. For such cases, we recommend selecting only the most effective stimuli from our publicly available stimulus set [47].

More limitations arise because these studies were not conducted in naturalistic settings. We do not know whether the effects of source reassignment would extend beyond a few seconds, or whether they would influence sounds encountered outside the lab. Future studies in clinical settings are needed. The generalizability of our studies is limited by our sample population of young adults, which should be remedied with broader sampling methods. While movies are not a practical treatment in a natural setting, descriptions of sources have the advantage of being available via memory and without any need for technology. Our stimuli were not customized triggers for each individual because custom movies are difficult to make. Addressing an individual’s unique trigger sounds may be easier with written descriptions. However, our written descriptions had a smaller effect than our movies. It is possible that further refinement of the written descriptions could improve both their matches and effectiveness.

It is worth noting that there are other factors that shift sound pleasantness. For example, self-generated perceptual input can also modulate emotional responses to sounds. Mimicking behavior (e.g., a listener sniffs in the presence of someone else who is sniffing) is observed in misophonia and it is speculated that this may reduce the severity of the negative experience from a misophonic trigger [48]. As another example, the emotional response to a soundscape, which typically involves multiple sound sources, depends upon the relative weight given to different sound sources, with unpleasant sounds being more influential than pleasant sounds [49].

Given that everyday sounds are ubiquitous, they can be difficult to avoid. A comprehensive treatment for misophonia will need to do more than block out external sounds or avoid situations that may involve triggering unpleasant sounds. The present findings could potentially be leveraged to help with everyday exposures to triggers. First, professional treatment could involve gathering a list of triggering sounds and finding plausible alternative sources for them. Alternative sources could be shown in movies such as the ones described in this research. If movies are unavailable, our data indicate that verbal descriptions of alternative sources should also be effective. This cognitive reframing could prepare the person to imagine an alternative source whenever they hear a trigger in the real world. If an individual can draw on that experience in real-time and imagine that trigger sounds are coming from a different source, this might reduce the severity of their emotional reaction to the sound in the moment.

## Summary and conclusion

Experiments 1, 3, and 5 revealed that movies displaying a visual source with a sound can robustly change the pleasantness of that sound. In Experiment 1, when a neutral visual source is paired with an unpleasant sound (*U*_*s*_*N*_*v*_), the unpleasant sound is rated as more pleasant than when the sound is paired with its original visual source (*U*_*s*_*U*_*v*_). In Experiment 3A, the effect is nearly equal and opposite for neutral sounds; the neutral sound is rated as less pleasant when paired with an unpleasant visual source (*N*_*s*_*U*_*v*_) than when the sound is paired with its original visual source (*N*_*s*_*N*_*v*_). The results of Experiments 1 and 5B indicate that the change in sound pleasantness from neutral visual sources is strongly influenced by the source plausibility match between the visual source and the paired sound. Specifically, a high match promotes the reassignment of the sound’s causal source. As Experiment 5A showed, visual pleasantness devoid of semantic content does not account for the effect of visual sources. In contrast, Experiment 2 shows that semantic content does account for the effect, because the written description of the neutral source events produces nearly as much of a change in sound pleasantness as the corresponding movies do. The effect of neutral visual sources is even more beneficial for misophonic than for non-misophonic participants.

In conclusion, attributing an unpleasant sound to a more neutral source may make the sounds more tolerable in the moment. We propose that a *change function* be used to determine whether a given stimulus is causing a source reassignment. Because an audio-visual match can mean multiple things, we propose that judgements about matching should be very clear about the definition of a match. Although movies produce a larger effect than words or images, presumably due to being more compelling, it is possible that purely semantic descriptions could be at least half as effective, while being much simpler to make and use. In the future, perhaps combining improved text descriptions with neutral or positive pictures would come close to being as effective as movies.

## Supporting information

S1 File Method details for baseline video pleasantness ratings. (DOCX)

S2 File Data file which contains average pleasantness and match ratings per sound across all experiments reported in this manuscript. (XLSX)

S1 Table Misophonic Trigger Literature Review. List of triggers includes experimental stimuli classified as Misophonic triggers, sounds and visuals with results providing they are triggers, self-reported triggers, triggers from Misophonic questionnaires, and case study triggers.
(DOCX)

S2 Table Demographics information: gender, age and ethnicity of participants in each experiment. (DOCX)

S3 Table Average identification accuracy and average misidentification rate for each neutral sound across all participants (*unscreened* group). ^a^Misidentification refers to confusing the sound for its planned unpleasant counterpart or for any other sound. ^b^Number of participants who misidentified the neutral sound for its planned, unpleasant counterpart. ^c^Number of participants who misidentified the neutral sound for a unpleasant source that was not the planned counterpart. ^d^Number of participants who misidentified the neutral sound for another neutral sound. ^e^Odds ratio is calculated by dividing the number of misidentifications per type (e.g., planned unpleasant source) by the number of total participants in the study. ^f^Odds ratio for the ‘other source’ category is calculated over a combined pool of the neutral and unpleasant instances.
(DOCX)

S1 Fig Experiment 1: *U*_*s*_*N*_*v*_ movie pleasantness versus match quality. The relationship between average sound pleasantness ratings for *U*_*s*_*N*_*v*_ pairs and average match quality ratings for *U*_*s*_*N*_*v*_ pairs in Experiment 1. The averages are calculated across two mutually exclusive participant groups: Misophonics (red squares), and Non-misophonics (gray circles). The 22 data points represent individual unpleasant sounds. The error bars reflect the standard error of the mean.
(TIF)

S2 Fig Experiment 1: *U*_*s*_*U*_*v*_ movie pleasantness versus match quality. The relationship between average sound pleasantness ratings for *U*_*s*_*U*_*v*_ pairs and average match quality ratings for *U*_*s*_*U*_*v*_ pairs in Experiment 1. The averages are calculated across two mutually exclusive participant groups: Misophonics (red squares), and Non-misophonics (gray circles). The 22 data points represent individual unpleasant sounds. The error bars reflect the standard error of the mean.
(TIF)

S3 Fig Experiment 1: Change function with respect to *U*_*s*_*U*_*v*_ match quality. The relationship between the change in average sound pleasantness ratings between the two audio-video conditions, and the average match quality ratings for *U*_*s*_*U*_*v*_ pairs in Experiment 1. The averages are calculated across two mutually exclusive participant groups: Misophonics (red squares), and Non-misophonics (gray circles). The changes are calculated by subtracting the average pleasantness rating the sound receives in *U*_*s*_*N*_*v*_ pairing from the rating the sound receives in *U*_*s*_*U*_*v*_ pairing. The 22 data points represent individual unpleasant sounds. The error bars reflect the standard error of the mean.
(TIF)

S4 Fig Data comparison between Experiment 1 and Samermit et al., (2022). (A) The relationship between average sound pleasantness ratings for *U*_*s*_*N*_*v*_ pairs versus average match quality ratings for *U*_*s*_*N*_*v*_ pairs in Experiment 1 and Samermit et al., (2022). Data from Experiment 1 (across all participants) is indicated by yellow symbols and a solid line. Note, our pleasantness ratings were transformed from an 11-point scale to a 5-point scale to be congruent with Samermit et al., (2022). Data from Samermit et al., (2022) is indicated by purple symbols and a dashed line. Yellow symbols with a purple outline reflect movies that were borrowed from Samermit et al., (2022) to be used in Experiment 1. Each data point represents the mean rating across observers for one unpleasant sound, while the error bar reflects the standard error of the mean. (B) The relationship between average sound pleasantness ratings for *U*_*s*_*U*_*v*_ pairs versus average match quality ratings for *U*_*s*_*U*_*v*_ pairs in Experiment 1 and Samermit et al., (2022). (C) The relationship between the change in average sound pleasantness ratings across the two pairs (*U*_*s*_*N*_*v*_ - *U*_*s*_*U*_*v*_) versus average match quality ratings for *U*_*s*_*N*_*v*_ pairs in Experiment 1 and Samermit et al., (2022). The 22 data points represent the mean change for each of the unpleasant sounds and the error bars reflect the standard error of the mean across participants.
(TIF)

S5 Fig Experiment 2: *U*_*s*_*N*_*D*_ movie pleasantness versus match quality. The relationship between average sound pleasantness ratings for *U*_*s*_*N*_*D*_ pairs and average match quality ratings for *U*_*s*_*N*_*D*_ pairs in Experiment 2. The averages are calculated across two mutually exclusive participant groups: Misophonics (red squares), and Non-misophonics (gray circles). The 22 data points represent individual unpleasant sounds. The error bars reflect the standard error of the mean.
(TIF)

S6 Fig Experiment 2: *U*_*s*_*U*_*D*_ movie pleasantness versus match quality. The relationship between average sound pleasantness ratings for *U*_*s*_*U*_*D*_ pairs and average match quality ratings for *U*_*s*_*U*_*D*_ pairs in Experiment 2. The averages are calculated across two mutually exclusive participant groups: Misophonics (red squares), and Non-misophonics (gray circles). The 22 data points represent individual unpleasant sounds. The error bars reflect the standard error of the mean.
(TIF)

S7 Fig Experiment 2: Change function with respect to *U*_*s*_*U*_*D*_ match quality. The relationship between the change in average sound pleasantness ratings between the two description conditions, and the average match quality ratings for *U*_*s*_*U*_*D*_ pairs in Experiment 2. The averages are calculated across two mutually exclusive participant groups: Misophonics (red squares), and Non-misophonics (gray circles). The changes are calculated by subtracting the average pleasantness rating the sound receives in *U*_*s*_*N*_*D*_ pairing from the rating the sound receives in *U*_*s*_*U*_*D*_ pairing. The 22 data points represent individual unpleasant sounds. The error bars reflect the standard error of the mean.
(TIF)

S8 Fig Experiment 3A: *N*_*s*_*U*_*v*_ movie pleasantness versus match quality. The relationship between average sound pleasantness ratings for *N*_*s*_*U*_*v*_ pairs and average match quality ratings for *N*_*s*_*U*_*v*_ pairs in Experiment 3A. The averages are calculated across all of the listeners in this *unscreened* group, irrespective of misophonic status. The 22 data points represent individual neutral sounds. The error bars reflect the standard error of the mean.
(TIF)

S9 Fig Experiment 3A: *N*_*s*_*N*_*v*_ movie pleasantness versus match quality. The relationship between average sound pleasantness ratings for *N*_*s*_*N*_*v*_ pairs and average match quality ratings for *N*_*s*_*N*_*v*_ pairs in Experiment 3A. The averages are calculated across all of the listeners in this *unscreened* group, irrespective of clinically significant misophonia status. The 22 data points represent individual neutral sounds. The error bars reflect the standard error of the mean.
(TIF)

S10 Fig Experiment 3A: Change function with respect to *N*_*s*_*N*_*v*_ match quality. The relationship between the change in average sound pleasantness ratings between the two audio-video conditions, and the average match quality ratings for *N*_*s*_*N*_*v*_ pairs in Experiment 3A. The averages are calculated across all listeners in this *unscreened* group, irrespective of misophonia status. The changes are calculated by subtracting the average pleasantness rating the sound receives in *N*_*s*_*U*_*v*_ pairing from the rating the sound receives in *N*_*s*_*N*_*v*_ pairing. The 22 data points represent individual neutral sounds. The error bars reflect the standard error of the mean.
(TIF)
