# Supplementary material for: Reassigning sources of misophonic trigger sounds to change their unpleasantness: Testing alternative mechanisms with a new set of movies, paintings, and words
Source: PLoS One. 2025 Apr 18;20(4):e0321594. doi: 10.1371/journal.pone.0321594 (PMC12007711; doi:10.1371/journal.pone.0321594)
Supplement: S1 File — (DOCX) [file pone.0321594.s001.docx]

# Baseline Video Ratings (Supplemental Materials)

To provide a baseline, we measured the pleasantness of the neutral and unpleasant silent video sources. The pleasantness of video sources are reported in Supplemental Data File S2.xlsx (“Baseline video pleasantness” sheet) and are used for ancillary data analysis in some of the main studies. In particular, we used these values to explore the impact of the pleasantness of the visual sources themselves, distinct from their effects on the source attribution of the sound. We report their correlation with the sound pleasantness changes associated with different video pairings. For example, in either Experiment 1 or 3A, participants could have not followed the instructions and could have rated visual pleasantness instead of sound pleasantness. Therefore, the pleasantness of the silent video tracks (e.g., *N_v_*) was examined in each study to test whether those ratings predict the pleasantness ratings of the sounds.

## Method

### **Participants**

Thirty-five participants (M_age_ = 23.54 years; range = 18 to 30 years; 16 females, 18 males, one non-binary) were tested irrespective of misophonia severity (after excluding 5 participants for failing catch trials). In this unscreened group, seven individuals (M_age_ = 22 years; range = 18 to 28 years; 2 females, 4 males, one non-binary) met our criteria for misophonia.

### **Stimuli**

The 44 silent visual sources were composed of our 22 unpleasant visual sources (*U_v_*), and 22 neutral visual sources (*N_v_*).

### **Procedure**

The study procedure had participants were shown silent video tracks and asked “how pleasant is the video?” They viewed a silent visual source (*U_v_* or *N_v_*, in random order) using an 11-point scale, wherein -5 indicated the video was very unpleasant, +5 indicated the video was very pleasant, and 0 indicated the video was neutral.

## Results

This study was conducted to assess the pleasantness of the silent video tracks (*N_v_* and *U_v_*). The silent, unpleasant visual sources (*U_v_*) were rated an average of -1.78 (range from -4.17 to 2.40) on the pleasantness scale, while the silent, neutral visual sources (*N_v_*) were rated an average of 1.29 (range -0.97 to 4.34). The mean ratings for all silent video tracks are in Supplemental data File S2.xls (“Baseline video pleasantness” sheet).

**Participant demographics.**

|  | |  | |  | Gender | | | | | | |  | |  | | | % Breakdown across Ethnicities | | | | | | | | | | | |
| --- | --- | --- | --- | --- | --- | --- | --- | --- | --- | --- | --- | --- | --- | --- | --- | --- | --- | --- | --- | --- | --- | --- | --- | --- | --- | --- | --- | --- |
| N | Status | | Group | | Female | | Male | | Non-  binary | | Prefer not to say | | Mean Age | | Age Range | White | | Asian | | Mixed | | Other | | Black | | Prefer not to say | |  |
| 7 | | Misophonic | | Unscreened | 2 | 4 | | 1 | | 0 | | 22 | | 18 - 28 | | | 60 | | 25.71 | | 2.86 | | 2.86 | | 8.57 | | 0 | |
| 28 | | Non-misophonic | | Unscreened | 14 | 14 | | 0 | | 0 | | 23.93 | | 19 - 30 | | |  |  |  |  |  |  |  |  |  |  |  |  |
