# Supplementary material for: Reassigning sources of misophonic trigger sounds to change their unpleasantness: Testing alternative mechanisms with a new set of movies, paintings, and words
Source: PLoS One. 2025 Apr 18;20(4):e0321594. doi: 10.1371/journal.pone.0321594 (PMC12007711; doi:10.1371/journal.pone.0321594)
Supplement: S1 Table — List of triggers includes experimental stimuli classified as Misophonic triggers, sounds and visuals with results providing they are triggers, self-reported triggers, triggers from Misophonic questionnaires, and case study triggers. (DOCX) [file pone.0321594.s003.docx]

**Table S1. Misophonic Trigger Literature Review.** List of triggers includes experimental stimuli classified as Misophonic triggers, sounds and visuals with results providing they are triggers, self-reported triggers, triggers from Misophonic questionnaires, and case study triggers.

| **Mode** | **Trigger** | **Labels** | **Sources** | **Count** |
| --- | --- | --- | --- | --- |
| Sound | General Chewing | “Eating”, Chewing 1”, “Chewing 2”, “Eating salad with cutlery sounds”, “Soft chewing”, “Eating sounds 1”, “Eating sounds 2”, “Chewing”, “Loud chewing”, “People eating”, “Eating”, “Chewing”, “Chewing with mouth open”, “Eating and slurping”, “Mouth open chewing”, “Sounds of eating”, “Chewing” | Savard et al. (2022)[1]; Enzler et al. (2021)[2] (THRICE); Hansen et al. (2021)[3]; Daniels et al. (2020)[4] (FOUR); Kumar et al. (2017, 2021)[5,6] (FOUR); Vitoratou et al. (2018)[7]; Wu et al. (2014)[8]; Edelstein et al. (2013)[9] (TWICE); Hadjipavlou et al. (2008)[10] (TWICE); Johnson et al. (2013)[11] (THRICE); Neal et al. (2013)[12]; Taylor (2017)[13]; Jastreboff and Jastreboff (2014)[14] (TWICE), Seaborne and Fiorella (2018)[15] | 31 |
|  | Human vocalizations | “Male chuckling”, “Baby crying”, “Singing”, “Humming”, “A child crying”, “Specific songs”, “High-pitched voices”, “Babies crying”, “High pitched or loud voices”, “Other people singing”, “One’s own voice”, “Specific type of laughter”, “Children’s voices”, “Screaming, crying babies”, “High-pitched voices”, “High-pitched voices/screams”, “Yawning”, “Whistling”, “People talking”, “Whispering”, “Laughing”, “Crying”, “Singing”, “Specific words” | Enzler et al. (2021) (TEN)[2]; Hansen et al. (2021)[3]; Vitoratou et al. (2018)[7]; Johnson et al. (2013)[11] (TWICE); Neal et al. (2013)[12]; Webber et al. (2014)[16] (TWICE); Taylor (2017)[13] (TWICE); Jastreboff and Jastreboff (2014)[14] (SIX) | 25 |
|  | General tapping noises | “Foot tapping”, “Finger tapping”, “Pacing”, “Pen tapping”, “Repetitive tapping”, “Footsteps”, “Tapping”, “Fingers tapping on the table”, “Tapping pencil on desk”, “Finger tapping”, “Cat walking on hardwood floor”, “Finger/nail tapping”, “Thumping”, “Leg shaking” | Enzler et al. (2021)[2] (FOUR); Vitoratou et al. (2018)[7] (FOUR); Wu et al. (2014)[8]; Edelstein et al. (2013)[9] (TWICE); Johnson et al. (2013)[11] (THRICE); Neal et al. (2013)[12]; Taylor (2017) (TWICE); Jastreboff and Jastreboff (2014)[14] | 18 |
|  | Metal Utensil / Dish noises | “Cutlery noises”, “Cutlery sounds”, “Eating salad with cutlery sounds”, “Knife cutting and rattiling”, “Utensils rubbing against plates”, “Cutlery clinking on plates”, “Sounds of surgical instruments in an operating room”, “Utensils hitting china”, “Cutlery and plates”, “Loading dishes in dishwasher”, “Fork hitting teeth”, “Cutlery”, “Fork/knife on plate” | Savard et al. (2022)[1]; Enzler et al. (2021)[2] (THRICE); Hansen et al. (2021)[3]; Daniels et al. (2020)[4] (TWICE); Kumar et al. (2017, 2021)[5,6] (TWICE); Johnson et al. (2013)[11]; Taylor (2017)[13]; Jastreboff and Jastreboff (2014)[14] (FOUR) | 17 |
|  | Dry / Crunchy Chewing | “Crunching chips”, “Apple crunching”, “Eating potato chips”, “Crisps eating”, “Crunching an apple”, “Crunching sounds”, “Biting/Crunching”, “Crunching of foods”, “Eating chips”, “Apple bite”, “Eating popcorn” | Enzler et al. (2021)[2] (THRICE); Hansen et al. (2021)[3] (TWICE); Daniels et al. (2020)[4] (TWICE); Kumar et al. (2017, 2021)[5,6] (TWICE); Vitoratou et al. (2018)[7]; Edelstein et al. (2013)[9]; Taylor (2017)[13]; Jastreboff and Jastreboff (2014)[14] | 15 |
|  | Sniffing | “Sniffling”, “Coughing/Sniffling” | Savard et al. (2022)[1]; Enzler et al. (2021)[2] (TWICE); Hansen et al. (2021)[3]; Daniels et al. (2020)[4]; Daniels et al. (2020)[4]; Kumar et al (2017, 2021)[5,6] (TWICE); Edelstein et al. (2013)[9]; Johnson et al. (2013)[11]; Taylor (2017)[13]; Jastreboff and Jastreboff (2014)[14] | 14 |
|  | Slurping | “Slurping”, “Slurping with eating”, “Sipping hot liquid” | Enzler et al. (2021)[2] (TWICE); Hansen et al. (2021)[3] (TWICE); Daniels et al. (2020)[4] (TWICE); Kumar et al (2017, 2021)[5,6] (TWICE); Vitoratou et al. (2018)[7]; Johnson et al. (2013)[11] (TWICE); Taylor (2017)[13]; | 14 |
|  | Mechanical - Humming /Rumbling sounds | “Car engine”, “Humming of object”, “Gas lamp flickering”, “Low-flying airplanes”, “Sound of a refrigerator”, “Supermarket freezer”, “Hum of electricity”, “Hum of a computer”, “Printer, copy machine, and fax sounds”, “Electric razor”, “Repetitive motor”, “Heater”, “Ventilation”, “Printer” | Enzler et al. (2021)[2] (THRICE); Hansen et al. (2021)[3] (TWICE); Vitoratou et al. (2018)[7] (TWICE); Jastreboff and Jastreboff (2014)[14] (TWICE); Jastreboff and Jastreboff (2014)[14] (FIVE); | 14 |
|  | General clicking noises | “Clicking a mouse”, “Gas stove lighter”, “Walking in heels”, “Empty bottles clanking in the distance”, “Clipping nails”, “Clinking glasses”, “Nail clipping”, “Clipping and filing fingernails”, “Mouse click”, “Nail clipping”, “Nail filing”, “Nail snapping” | Enzler et al. (2021)[2] (FOUR); Hansen et al. (2021)[3] (FIVE); Taylor (2017)[13]; Taylor (2017)[13]; Jastreboff and Jastreboff (2014)[14] | 12 |
|  | General mouth (tongue and lip) sounds | “Unspecified mouth sounds”, “Tongue clicking/licking”, “Saliva/spitting”, “Lip smacking”, “People licking their lips” | Enzler et al. (2021)[2] (FOUR); Vitoratou et al. (2018)[7]; Edelstein et al. (2013)[9]; Hadjipavlou et al. (2008)[10]; Johnson et al. (2013)[11] (TWICE); Taylor (2017)[13]; Jastreboff and Jastreboff (2014)[14] | 11 |
|  | Squeaking sounds | “Windshield wipers”, “Styrofoam rubbing together”, “Bus brakes”, “Sound of car brakes or motorcycle brakes”, “Chalk on a blackboard”, “Sound of drawing with a felt tip pen”, “Squeaking guitar strings”, “Squeaky door”, “Chalk blackboard” | Enzler et al. (2021)[2] (FOUR); Hansen et al. (2021)[3] (TWICE); Johnson et al. (2013)[11]; Taylor (2017)[13]; Jastreboff and Jastreboff (2014)[14] (THRICE); | 11 |
|  | Animal vocalizations | “Repetitive barking”, “Dogs barking”, “Rooster”, “Cats”, “Birds”, “Birds chirping”, “Crow cawing”, “Frog croaking”, | Enzler et al. (2021)[2] (FOUR); Hansen et al. (2021)[3] (THRICE); Vitoratou et al. (2018)[7]; Edelstein et al. (2013)[9]; Taylor (2017)[13]; Jastreboff and Jastreboff (2014)[14]; | 11 |
|  | Rustling sounds | “Packet rustling”, “Paper compaction”, “Turning pages”, “Bags of chips”, “Packet opening and eating”, “Rustling plastic”, “Rustling paper”, “Rustling”, “Plastic bags”, “Crinkly bags”, “Crumpling or wrinkling paper”, “Crumpling of papers or wrappers” | Savard et al. (2022)[1]; Enzler et al. (2021)[2] (THRICE); Daniels et al. (2020)[4]; Vitoratou et al. (2018)[7] (TWICE); Wu et al. (2014)[8]; Edelstein et al. (2013)[9]; Jastreboff and Jastreboff (2014)[14]; Seaborne and Fiorella (2018)[15] | 10 |
|  | Whirring mechanical noises | “Sounds of a dental procedure”, “Drilling”, “Power tools”, “Leaf blower”, “Lawn mower”, “Swimming pool pump”, “Vacuum cleaner”, “Garbage disposal”, “Hairdryer”, “Construction work” | Enzler et al. (2021)[2]; Jastreboff and Jastreboff (2014)[14] (NINE); | 10 |
|  | Swallowing | “Swallowing”, “Gulping water” | Enzler et al. (2021)[2] (TWICE); Hansen et al. (2021)[3]; Daniels et al. (2020)[4]; Kumar et al (2017, 2021)[5,6]; Vitoratou et al. (2018)[7]; Taylor (2017)[13]; Jastreboff and Jastreboff (2014)[14]; | 9 |
|  | General Breathing | “Breathing”, “Breathing sound”, “Mouth breathing”, “Other person breathing” | Enzler et al. (2021)[2]; Hansen et al. (2021)[3]; Hansen et al. (2021)[3]; Daniels et al. (2020)[4]; Kumar et al (2017, 2021)[5,6]; Vitoratou et al. (2018)[7]; Johnson et al. (2013)[11]; Jastreboff and Jastreboff (2014)[14] | 9 |
|  | General language noises | “Certain letter sounds”, “Certain accents”, “Vowel/consonant sounds”, “Speaking”, “Certain speech patterns”, “Conversations and murmuring at various volumes”, “Certain words”, “Repetitive words” | Enzler et al. (2021)[2]; Vitoratou et al. (2018)[7] (TWICE); Wu et al. (2014)[8]; Hadjipavlou et al. (2008)[10] (TWICE); Johnson et al. (2013) (THRICE)[11] | 9 |
|  | Atmosphere / background sounds | “Plane”, “Church bell”, “Crowded party”, “Car”, “Motorbike”, “Noisy neighbors”, “Street sounds”, “Sounds of driving on street or highway”, “Neighbors playing television”, “Neighbors playing music” | Enzler et al. (2021)[2] (SEVEN); Taylor (2017)[13]; Jastreboff and Jastreboff (2014)[14] (TWICE); | 9 |
|  | Coughing | “Coughing”, “Cough”, “Coughing/Sniffling” | Savard et al. (2022)[1]; Enzler et al. (2021)[2] (TWICE); Hansen et al. (2021)[3]; Daniels et al. (2020)[4]; Kumar et al (2017, 2021)[5,6]; Vitoratou et al. (2018)[7] | 8 |
|  | Water noises | “Light splashing of water”, “Boiling water”, “Water drops”, “Washing hands”, “Water leak”, “Water running, “Rain”, “Water dripping”, | Enzler et al. (2021)[2] (FIVE); Hansen et al. (2021)[3] (TWICE); Jastreboff and Jastreboff (2014)[14]; | 8 |
|  | Keyboard typing | “Keyboard”, “Typing”, “Keyboard tapping”, “Typing on a keyboard” | Enzler et al. (2021)[2] (TWICE); Hansen et al. (2021)[3]; Vitoratou et al. (2018)[7]; Edelstein et al. (2013)[9]; Taylor (2017)[13]; Jastreboff and Jastreboff (2014)[14]; | 7 |
|  | Siren/Alarms | “Ambulances”, “Car alarms”, “Loud rings”, “School bell”, “Warning sounds (e.g., sirens, car horns, beepers)”, “Horn”, “Siren” | Enzler et al. (2021)[2] (TWICE); Neal et al. (2013)[12] (TWICE); Jastreboff and Jastreboff (2014)[14] (THRICE); | 7 |
|  | Pen clicking | “Pen click”, “Clicking a pen, “Clicking pen”, “Pen clicking” | Enzler et al. (2021)[2] (TWICE); Hansen et al. (2021)[3]; Vitoratou et al. (2018)[7]; Edelstein et al. (2013)[9]; Taylor (2017)[13] | 6 |
|  | Throat clearing | “Throat clearing”; “Clearing throat” | Enzler et al. (2021)[2] (TWICE); Hansen et al. (2021)[3] (TWICE); Vitoratou et al. (2018)[7]; Taylor (2017)[13] | 6 |
|  | Hard Breathing | “Hard breathing”, “Breath running”, “Loud breathing”, “Heavy breathing”, “Wheezing” | Enzler et al. (2021)[2] (THRICE); Johnson et al. (2013)[11]; Taylor (2017)[13] | 5 |
|  | Snoring | “Snoring”, “Other person in bed, snoring” | Enzler et al. (2021)[2] (TWICE); Hansen et al. (2021)[3]; Johnson et al. (2013)[11]; Jastreboff and Jastreboff (2014)[14] | 5 |
|  | Friction noises | “Clothes friction”, “Headphone friction”, “Hands friction”, “Scuffing shoes”, “Shuffling of feet” | Enzler et al. (2021)[2] (FOUR); Johnson et al. (2013)[11] | 5 |
|  | Random mouth vocalization sounds | “Gargling”, “Gurling”, “Burping, “Gagging” | Enzler et al. (2021)[2] (FOUR) | 4 |
|  | Swinging or sliding noises | “Swinging on a swingset”, “Swinging legs”, “Foot wiggling”, “Sliding door” | Enzler et al. (2021)[2]; Hansen et al. (2021); Vitoratou et al. (2018)[7] (TWICE) | 4 |
|  | Sudden noises | “Slamming doors”, “Sudden sounds (e.g., object dropped on hard surface)”, “Cymbal”, “Door slamming” | Enzler et al. (2021)[2] (TWICE); Jastreboff and Jastreboff (2014)[14] (TWICE) | 4 |
|  | Clock noises | “Clock ticking”, “Clock tick” | Enzler et al. (2021)[2]; Hansen et al. (2021)[3]; Vitoratou et al. (2018)[7]; Edelstein et al. (2013)[9] | 4 |
|  | Music-related | “Repetitive music”, “Musical instruments”, “Instruments”, “Wind chimes” | Enzler et al. (2021)[2] (TWICE); Hansen et al. (2021)[3]; Jastreboff and Jastreboff (2014)[14]; | 4 |
|  | Wet / Sloppy Chewing | “Chewing gum” | Hansen et al. (2021)[3]; Seaborne and Fiorella (2018)[15] | 3 |
|  | Sneezing | “Sneezing”, “Blowing nose” | Enzler et al. (2021)[2] (TWICE); Hansen et al. (2021)[3] | 3 |
|  | Bouncing a ball | “Bouncing a basketball”, “Basketball dribbling”, “Football” | Enzler et al. (2021)[2]; Hansen et al. (2021)[3] (TWICE) | 3 |
|  | Body picking noises | “Nail biting”, “People picking their nails” | Enzler et al. (2021)[2]; Vitoratou et al. (2018)[7]; Hadjipavlou et al. (2008)[10] | 3 |
|  | Scratching noises | “Nails scratching against the scalp”, “Metal scratching”, “Nails scratching” | Enzler et al. (2021)[2] (TWICE); Johnson et al. (2013)[11] | 3 |
|  | Clattering noises | “Train on a track, particularly under a bridge”, “Keys rattling”, “Keys” | Enzler et al. (2021)[2]; Jastreboff and Jastreboff (2014)[14] (TWICE) | 3 |
|  | Popping or cracking sounds | “Finger snapping/crackling”, “Popping popcorn”, “Bones cracking” | Enzler et al. (2021)[2] (TWICE); Jastreboff and Jastreboff (2014)[14] | 3 |
|  | Writing | “Writing”, “Writing on table” | Enzler et al. (2021)[2]; Hansen et al. (2021)[3] | 2 |
|  | Low Frequency sounds | “Low frequency bass sounds”, | Vitoratou et al. (2018)[7]; Edelstein et al. (2013)[9] | 2 |
|  | Whistling noises | “Whistling sound”, “Whistling sounds” | Vitoratou et al. (2018)[7]; Edelstein et al. (2013)[9] | 2 |
|  | Sucking teeth | “Teeth sucking”, “Sucking teeth” | Enzler et al. (2021)[2]; Johnson et al. (2013)[11] | 2 |
|  | Announcements | “Announcements in a metro, train station, airport, or supermarket”, “Announcement on airplane or train” | Jastreboff and Jastreboff (2014)[14] (TWICE) | 2 |
|  | Tooth brushing | “Toothbrush”, “Teeth brushing” | Enzler et al. (2021)[2]; Jastreboff and Jastreboff (2014)[14] | 2 |
|  | Miscellaneous nasal sounds | “Nose whistling”, “Snorting”, | Enzler et al. (2021)[2] (TWICE) | 2 |
|  | Chopping noises | “Chopping vegetables” | Hansen et al. (2021)[3] | 1 |
|  | Hammering | “Hammering” | Hansen et al. (2021)[3] | 1 |
|  | Hiccuping | “Hiccups” | Vitoratou et al. (2018)[7] | 1 |
|  | Fire noises | “Daytime forest bonfire” | Hansen et al. (2021)[3] | 1 |
|  | Scraping | “Shoveling cement” | Jastreboff and Jastreboff (2014)[14] | 1 |
|  | Toilet | “Flushing toilet” | Jastreboff and Jastreboff (2014)[14] | 1 |
|  | Rubbing sounds | “Moving hand on a surface” | Jastreboff and Jastreboff (2014)[14] | 1 |
|  | Grinding teeth | “Teeth friction” | Enzler et al. (2021)[2] | 1 |
|  | Clapping | “Clapping” | Enzler et al. (2021)[2] | 1 |
|  | Wind noises | “Wind howling”, | Hansen et al. (2021)[3] | 1 |
| Visual | Moving Body parts | “Others shaking their foot around”, “Others moving toes/fingers”, “Someone jiggling or swinging their leg”, “Someone shaking their foot”, | Johnson et al. (2013)[11] (THRICE); Taylor (2017)[13] (TWICE) | 5 |
|  | Seeing lips moving/ eating / speaking | “Seeing someone chew gum”, “Lips moving”, “The sight of lips moving”, | Vitoratou et al. (2018)[7]; Hadjipavlou et al. (2008)[10]; Taylor (2017)[13] (TWICE) | 4 |
|  | Hair twirling | “Hair twirling”, “People playing with their hair”, “Someone twirling their hair” | Vitoratou et al. (2018)[7]; Johnson et al. (2013)[11]; Taylor (2017)[13] | 3 |
|  | Having hands around mouth | “Hands to mouth” | Vitoratou et al. (2018)[7] | 1 |
|  | Textures | “Slimy textures” | Vitoratou et al. (2018)[7] | 1 |
|  | Skin picking | “Skin picking” | Vitoratou et al. (2018)[7] | 1 |

*Note.* Some labels were excluded for being too vague (Nasal sounds, mechanical/motor sounds, toothbrush, belt buckle, electronic cigarette, etc) or for being too specific.

**References**

1. Savard M-A, Sares AG, Coffey EBJ, Deroche MLD. Specificity of Affective Responses in Misophonia Depends on Trigger Identification. Front Neurosci. 2022;16: 879583. doi:10.3389/fnins.2022.879583

2. Enzler F, Loriot C, Fournier P, Noreña AJ. A psychoacoustic test for misophonia assessment. Sci Rep. 2021;11: 11044. doi:10.1038/s41598-021-90355-8

3. Hansen HA, Leber AB, Saygin ZM. What sound sources trigger misophonia? Not just chewing and breathing. J Clin Psychol. 2021;77: 2609–2625. doi:10.1002/jclp.23196

4. Daniels EC, Rodriguez A, Zabelina DL. Severity of misophonia symptoms is associated with worse cognitive control when exposed to misophonia trigger sounds. PLOS ONE. 2020;15: e0227118. doi:10.1371/journal.pone.0227118

5. Kumar S, Tansley-Hancock O, Sedley W, Winston JS, Callaghan MF, Allen M, et al. The Brain Basis for Misophonia. Current Biology. 2017;27: 527–533. doi:10.1016/j.cub.2016.12.048

6. Kumar S, Dheerendra P, Erfanian M, Benzaquén E, Sedley W, Gander PE, et al. The Motor Basis for Misophonia. J Neurosci. 2021;41: 5762–5770. doi:10.1523/JNEUROSCI.0261-21.2021

7. Vitoratou S. The S-Five:a psychometric tool for assessing misophonia. PsyArXiv; 2018. doi:10.31234/osf.io/fqbm3

8. Wu MS, Lewin AB, Murphy TK, Storch EA. Misophonia: Incidence, Phenomenology, and Clinical Correlates in an Undergraduate Student Sample: Misophonia. J Clin Psychol. 2014;70: 994–1007. doi:10.1002/jclp.22098

9. Edelstein M, Brang D, Rouw R, Ramachandran VS. Misophonia: physiological investigations and case descriptions. Front Hum Neurosci. 2013;7. doi:10.3389/fnhum.2013.00296

10. Hadjipavlou G, Baer S, Lau A, Howard A. SELECTIVE SOUND INTOLERANCE AND EMOTIONAL DISTRESS: WHAT EVERY CLINICIAN SHOULD HEAR. Psychosomatic Medicine. 2008;70: 739–740. doi:10.1097/PSY.0b013e318180edc2

11. Johnson PL, Webber TA, Wu MS, Lewin AB, Murphy TK, Storch EA. When selective audiovisual stimuli become unbearable: a case series on pediatric misophonia. Neuropsychiatry. 2013;3: 569–575. doi:10.2217/npy.13.70

12. Neal M, Cavanna AE. Selective Sound Sensitivity Syndrome (Misophonia) in a Patient With Tourette Syndrome. JNP. 2013;25: E01–E01. doi:10.1176/appi.neuropsych.11100235

13. Taylor S. Misophonia: A new mental disorder? Medical Hypotheses. 2017;103: 109–117. doi:10.1016/j.mehy.2017.05.003

14. Jastreboff P, Jastreboff M. Treatments for Decreased Sound Tolerance (Hyperacusis and Misophonia). Semin Hear. 2014;35: 105–120. doi:10.1055/s-0034-1372527

15. Seaborne A, Fiorella L. Effects of background chewing sounds on learning: The role of misophonia sensitivity. Applied Cognitive Psychology. 2018;32: 264–269. doi:10.1002/acp.3387

16. Webber TA, Johnson PL, Storch EA. Pediatric misophonia with comorbid obsessive–compulsive spectrum disorders. General Hospital Psychiatry. 2014;36: 231.e1-231.e2. doi:10.1016/j.genhosppsych.2013.10.018
