# Supplementary material for: Reassigning sources of misophonic trigger sounds to change their unpleasantness: Testing alternative mechanisms with a new set of movies, paintings, and words
Source: PLoS One. 2025 Apr 18;20(4):e0321594. doi: 10.1371/journal.pone.0321594 (PMC12007711; doi:10.1371/journal.pone.0321594)
Supplement: S2 Table — (DOCX) [file pone.0321594.s004.docx]

| **Table S2** |  |  |  |  |  |  |  |  |  |  |  |  |  |  |  |
| --- | --- | --- | --- | --- | --- | --- | --- | --- | --- | --- | --- | --- | --- | --- | --- |
| *Demographics information: gender, age, and ethnicity of participants in each experiment* | | | | | | | |  |  |  |  |  |  |  |  |
|  |  |  |  |  |  |  |  |  |  |  |  |  |  |  |  |
|  |  |  |  | Gender | | | |  |  | % Breakdown across Ethnicities | | | | | |
| Experiment | N | Status | Group | Female | Male | Non-  binary | Prefer not to say | Mean Age | Age Range | White | Asian | Mixed | Other | Black | Prefer not to say |
| 1 | 20 | Misophonic | Misophonic | 8 | 10 | 2 | 0 | 24.75 | 18 - 36 | 19.15 | 31.91 | 12.77 | 6.38 | 8.51 | 21.28 |
|  | 62 | Non-misophonic | Control | 34 | 28 | 0 | 0 | 24.48 | 18 - 35 |  |  |  |  |  |  |
| 2 | 26 | Misophonic | Misophonic | 16 | 6 | 4 | 0 | 22.27 | 18 - 29 | 25.93 | 34.57 | 7.41 | 9.88 | 3.7 | 18.52 |
|  | 55 | Non-misophonic | Control | 18 | 35 | 1 | 1 | 21.24 | 18 - 30 |  |  |  |  |  |  |
| 3A | 6 | Misophonic | Unscreened | 4 | 2 | 0 | 0 | 21.83 | 19 - 28 | 41.18 | 29.41 | 8.82 | 13.24 | 1.47 | 5.88 |
|  | 62 | Non-misophonic | Unscreened | 31 | 29 | 2 | 0 | 21.68 | 18 - 30 |  |  |  |  |  |  |
| 3B | 1 | Misophonic | Unscreened | 1 | 0 | 0 | 0 | 20 | NA | 5.88 | 47.06 | 11.76 | 17.65 | 5.88 | 11.76 |
|  | 16 | Non-misophonic | Unscreened | 9 | 7 | 0 | 0 | 18.94 | 18 - 20 |  |  |  |  |  |  |
| 3C | 3 | Misophonic | Unscreened | 3 | 0 | 0 | 0 | 19.67 | 18 - 22 | 53.13 | 34.38 | 0 | 0 | 9.38 | 3.13 |
|  | 29 | Non-misophonic | Unscreened | 12 | 13 | 4 | 0 | 22.1 | 18 - 29 |  |  |  |  |  |  |
| 4 | 11 | Misophonic | Unscreened | 5 | 6 | 0 | 0 | 25.18 | 18 - 29 | 53.13 | 9.38 | 15.63 | 0 | 9.38 | 12.5 |
|  | 21 | Non-misophonic | Unscreened | 7 | 14 | 0 | 0 | 24 | 18 - 30 |  |  |  |  |  |  |
| 5A | 4 | Misophonic | Unscreened | 3 | 1 | 0 | 0 | 20 | 18 - 22 | 50 | 35 | 0 | 15 | 0 | 0 |
|  | 16 | Non-misophonic | Unscreened | 14 | 1 | 1 | 0 | 20 | 18 - 22 |  |  |  |  |  |  |
| 5B | 5 | Misophonic | Unscreened | 4 | 1 | 0 | 0 | 19.4 | 18 - 20 | 20.59 | 55.89 | 0 | 14.71 | 5.88 | 2.94 |
|  | 29 | Non-misophonic | Unscreened | 19 | 10 | 0 | 0 | 19.76 | 18 - 31 |  |  |  |  |  |  |
| 5C | 3 | Misophonic | Unscreened | 3 | 0 | 0 | 0 | 19.67 | 18 - 22 | 53.13 | 34.38 | 0 | 0 | 9.38 | 3.13 |
|  | 29 | Non-misophonic | Unscreened | 12 | 13 | 4 | 0 | 22.1 | 18 - 29 |  |  |  |  |  |  |
| Note. Our recruitment process for Experiments 3A-C, 4, 5A-C did not actively recruit or seek out misophonic participants, instead all participants were included. We refer to this population of participants as the “Unscreened” group. These experiments (i.e., 3A-C, 4, 5A-C) provided an estimate of the prevalence of misophonic listeners in our local population. | | | | | | | | | | | | | | | |
| ‘Mixed’ category refers to the individual identifying with more than one ethnicity (e.g., White and Asian). | | | | | | | | |  |  |  |  |  |  |  |
| ‘Other’ category refers to the individual identifying as either Middle Eastern, Native American or some other ethnicity that was not listed. | | | | | | | | | | |  |  |  |  |  |
