# Supplementary material for: Reassigning sources of misophonic trigger sounds to change their unpleasantness: Testing alternative mechanisms with a new set of movies, paintings, and words
Source: PLoS One. 2025 Apr 18;20(4):e0321594. doi: 10.1371/journal.pone.0321594 (PMC12007711; doi:10.1371/journal.pone.0321594)
Supplement: S3 Table — aMisidentification refers to confusing the sound for its planned unpleasant counterpart or for any other sound. bNumber of participants who misidentified the neutral sound for its planned, unpleasant counterpart. cNumber of participants who misidentified the neutral sound for a unpleasant source that was not the planned counterpart. dNumber of participants who misidentified the neutral sound for another neutral sound. eOdds ratio is calculated by dividing the number of misidentifications per type (e.g., planned unpleasant source) by the number of total participants in the study. fOdds ratio for the ‘other source’ category is calculated over a combined pool of the neutral and unpleasant instances. (DOCX) [file pone.0321594.s005.docx]

**Table S3. Average identification accuracy and average misidentification rate for each neutral sound across all participants (Unscreened group).**

|  |  |  | **Misidentification Instances** | | |
| --- | --- | --- | --- | --- | --- |
|  |  |  | *Neutral sources* | | *Unpleasant source* |
| **Sound Name** | **Identification Accuracy (%)** | **Misidentification^a^ Rate (%)** | **Planned source^b^ [1]** | **Other source^c^ [42]** | **Other source^d^** |
| Pulling tape on/off dispenser | 78.1 | 21.9 | 2 | 1 | 4 |
| Lawn sprinkler | 90.6 | 9.4 | 2 | 0 | 1 |
| Shaking a bottle of beads | 96.9 | 3.1 | 1 | 0 | 0 |
| Campfire burning | 100.0 | 0.0 | 0 | 0 | 0 |
| Snapping a stick | 43.8 | 56.3 | 10 | 6 | 2 |
| Scraping ruler | 59.4 | 40.6 | 0 | 3 | 10 |
| Birds chirping | 100.0 | 0.0 | 0 | 0 | 0 |
| Pulling facial tissues | 100.0 | 0.0 | 0 | 0 | 0 |
| Twisting Rubik's cube | 75.0 | 25.0 | 2 | 0 | 6 |
| Releasing a measuring tape | 84.4 | 15.6 | 0 | 1 | 3 |
| Tapping bag on tambourine | 100.0 | 0.0 | 0 | 0 | 0 |
| Stirring noodle soup | 87.5 | 12.5 | 0 | 1 | 3 |
| Ducks splashing | 93.8 | 6.3 | 0 | 0 | 2 |
| Deer munching | 62.5 | 37.5 | 0 | 5 | 7 |
| Bubbles rising | 90.6 | 9.4 | 2 | 1 | 0 |
| Pumping air | 53.1 | 46.9 | 11 | 3 | 1 |
| Dragging broom across table | 71.9 | 28.1 | 0 | 1 | 8 |
| Spray bottle | 84.4 | 15.6 | 0 | 3 | 2 |
| Ripping fabric | 96.9 | 3.1 | 0 | 0 | 1 |
| Releasing air from balloon | 46.9 | 53.1 | 2 | 5 | 10 |
| Stream flowing | 100.0 | 0.0 | 0 | 0 | 0 |
| Bouncing ball | 96.9 | 3.1 | 0 | 0 | 1 |
| **Total number of misidentifications** | **580** |  | **32** | **61** | **30** |
|  |  |  |  |  |  |
|  |  |  |  |  |  |
| **Odds Ratio^e^** | **18.125** |  | **1.00** | **0.07** | |
|  |  |  |  |  |  |

^a^Misidentification refers to confusing the sound for its planned unpleasant counterpart or for any other sound.

^b^Number of participants who misidentified the neutral sound for its planned, unpleasant counterpart.

^c^Number of participants who misidentified the neutral sound for a unpleasant source that was not the planned counterpart.

^d^Number of participants who misidentified the neutral sound for another neutral sound.

^e^Odds ratio is calculated by dividing the number of misidentifications per type (e.g., planned unpleasant source) by the number of total participants in the study.

^f^Odds ratio for the ‘other source’ category is calculated over a combined pool of the neutral and unpleasant instances.
