# Supplementary figures and images for: Reassigning sources of misophonic trigger sounds to change their unpleasantness: Testing alternative mechanisms with a new set of movies, paintings, and words
Source: PLoS One. 2025 Apr 18;20(4):e0321594. doi: 10.1371/journal.pone.0321594 (PMC12007711; doi:10.1371/journal.pone.0321594)

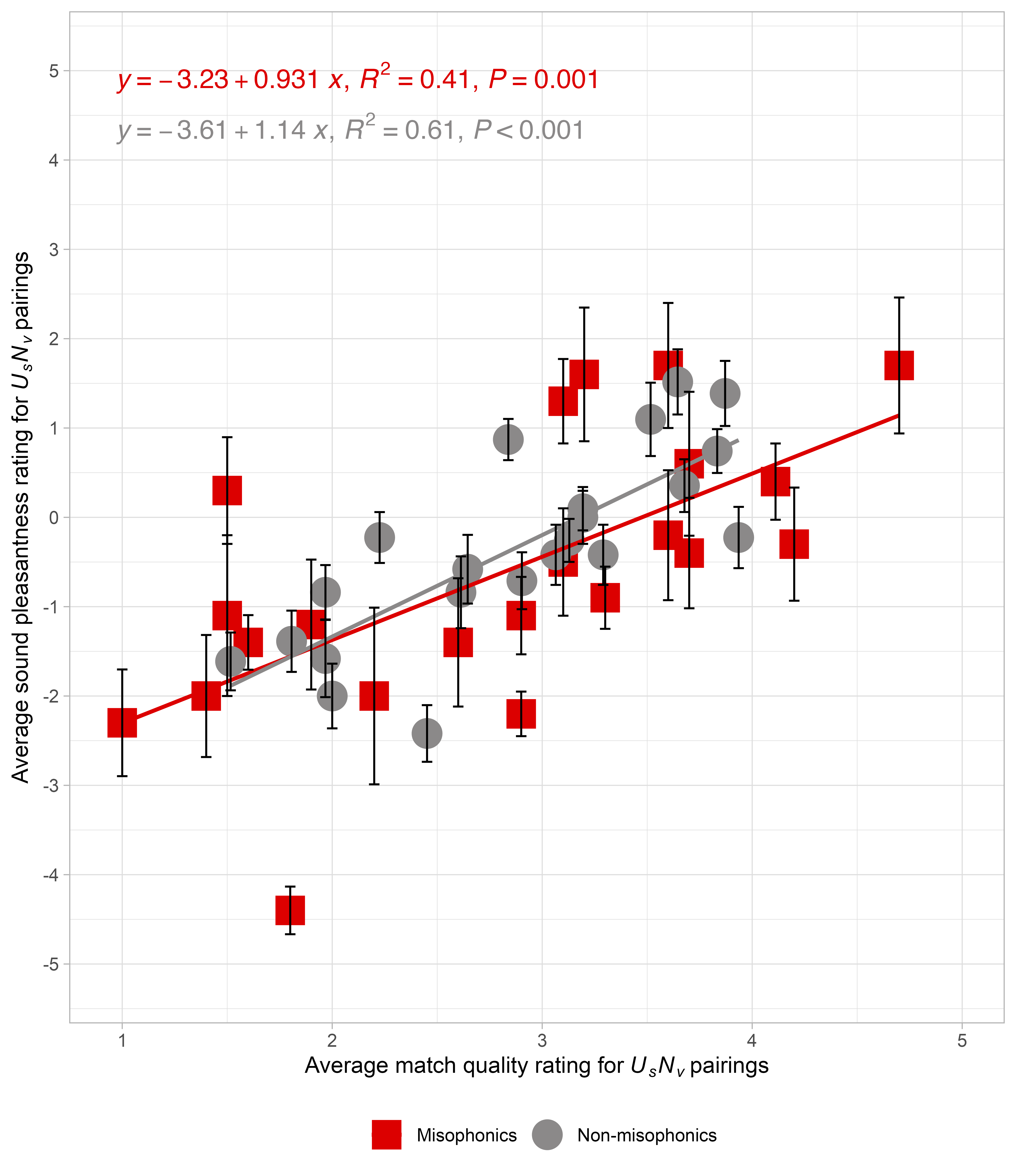

Supplement: S1 Fig — The relationship between average sound pleasantness ratings for UsNv pairs and average match quality ratings for UsNv pairs in Experiment 1. The averages are calculated across two mutually exclusive participant groups: Misophonics (red squares), and Non-misophonics (gray circles). The 22 data points represent individual unpleasant sounds. The error bars reflect the standard error of the mean. (TIF) [file pone.0321594.s006.tif]

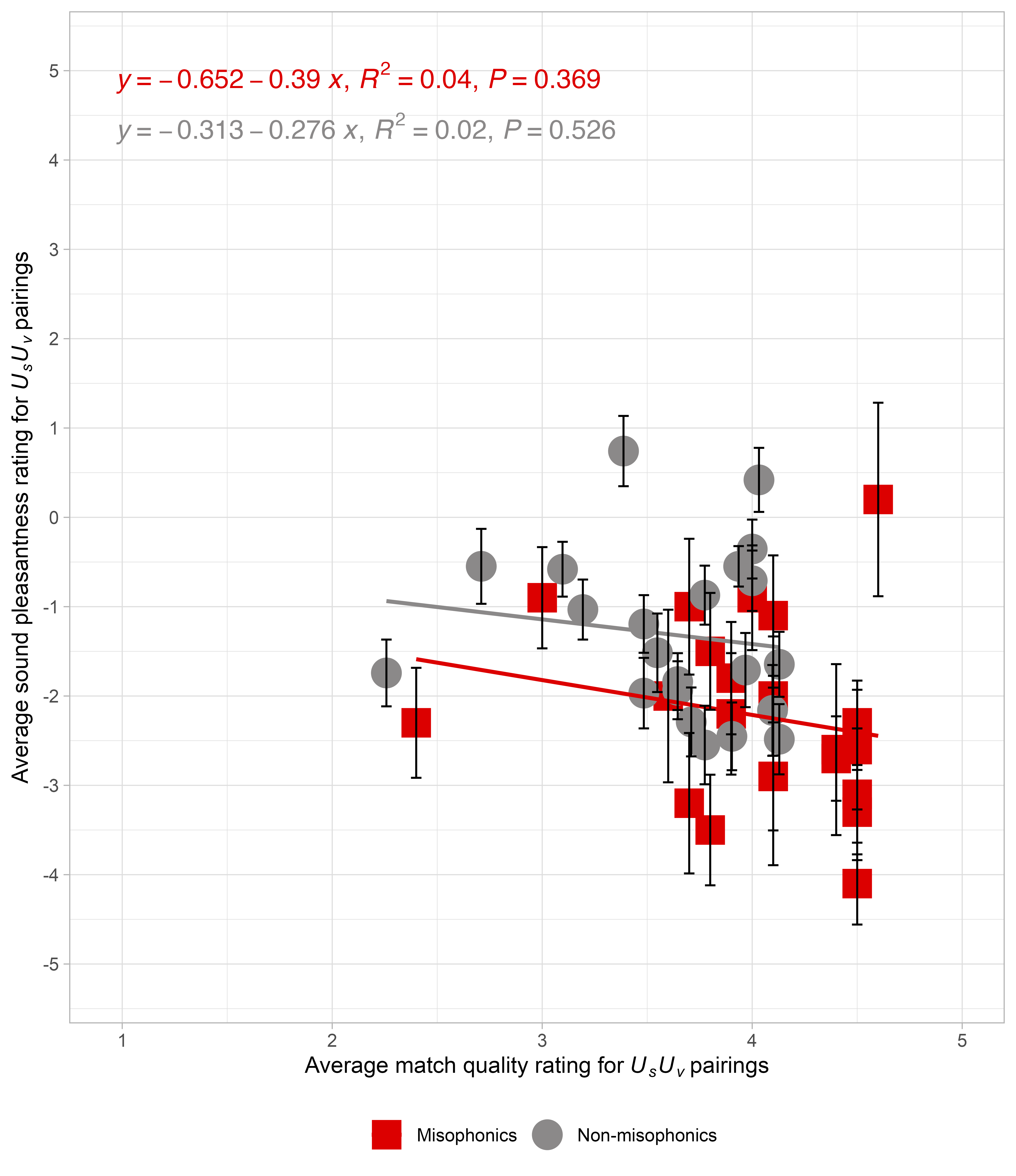

Supplement: S2 Fig — The relationship between average sound pleasantness ratings for UsUv pairs and average match quality ratings for UsUv pairs in Experiment 1. The averages are calculated across two mutually exclusive participant groups: Misophonics (red squares), and Non-misophonics (gray circles). The 22 data points represent individual unpleasant sounds. The error bars reflect the standard error of the mean. (TIF) [file pone.0321594.s007.tif]

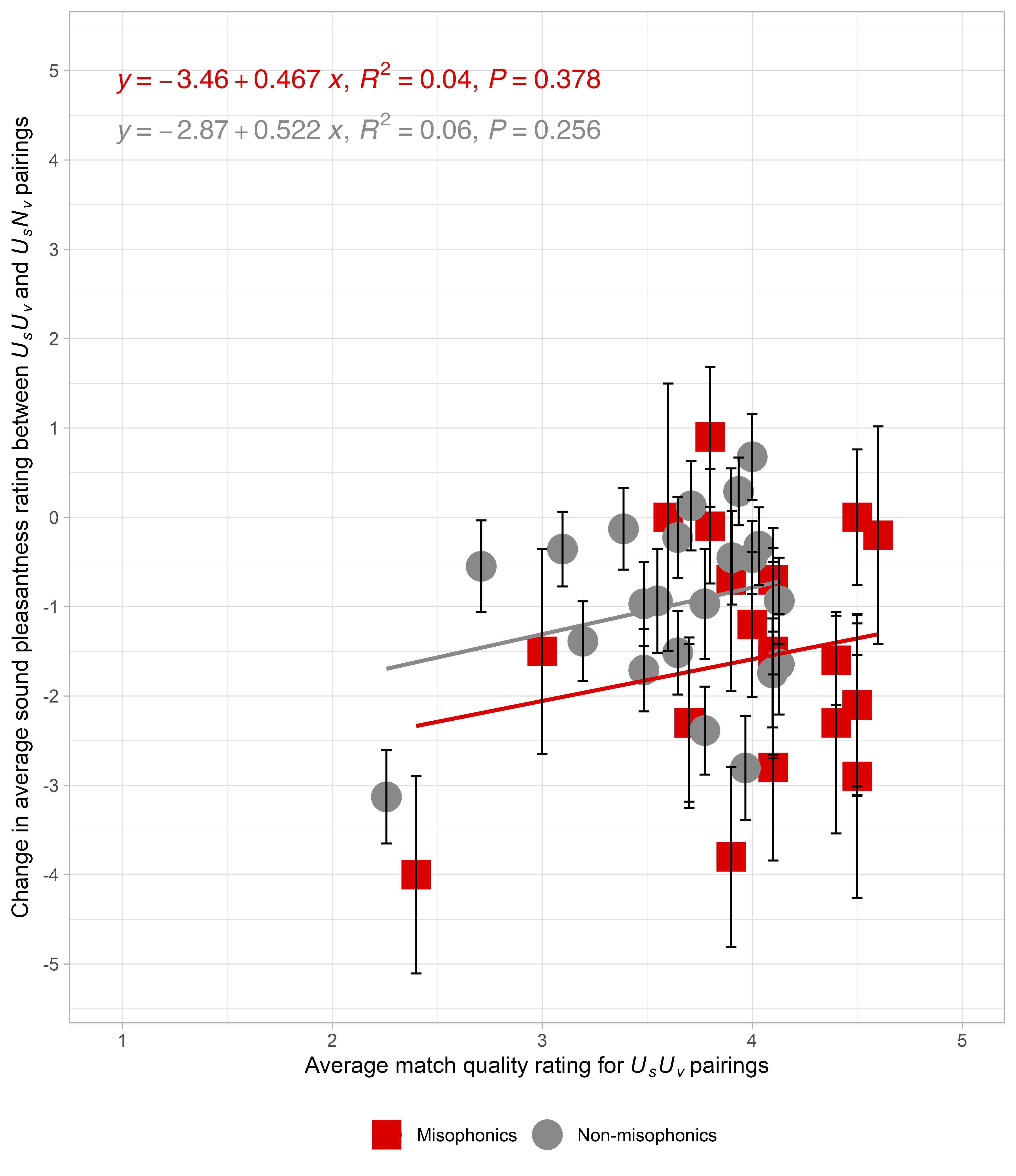

Supplement: S3 Fig — The relationship between the change in average sound pleasantness ratings between the two audio-video conditions, and the average match quality ratings for UsUv pairs in Experiment 1. The averages are calculated across two mutually exclusive participant groups: Misophonics (red squares), and Non-misophonics (gray circles). The changes are calculated by subtracting the average pleasantness rating the sound receives in UsNv pairing from the rating the sound receives in UsUv pairing. The 22 data points represent individual unpleasant sounds. The error bars reflect the standard error of the mean. (TIF) [file pone.0321594.s008.tif]

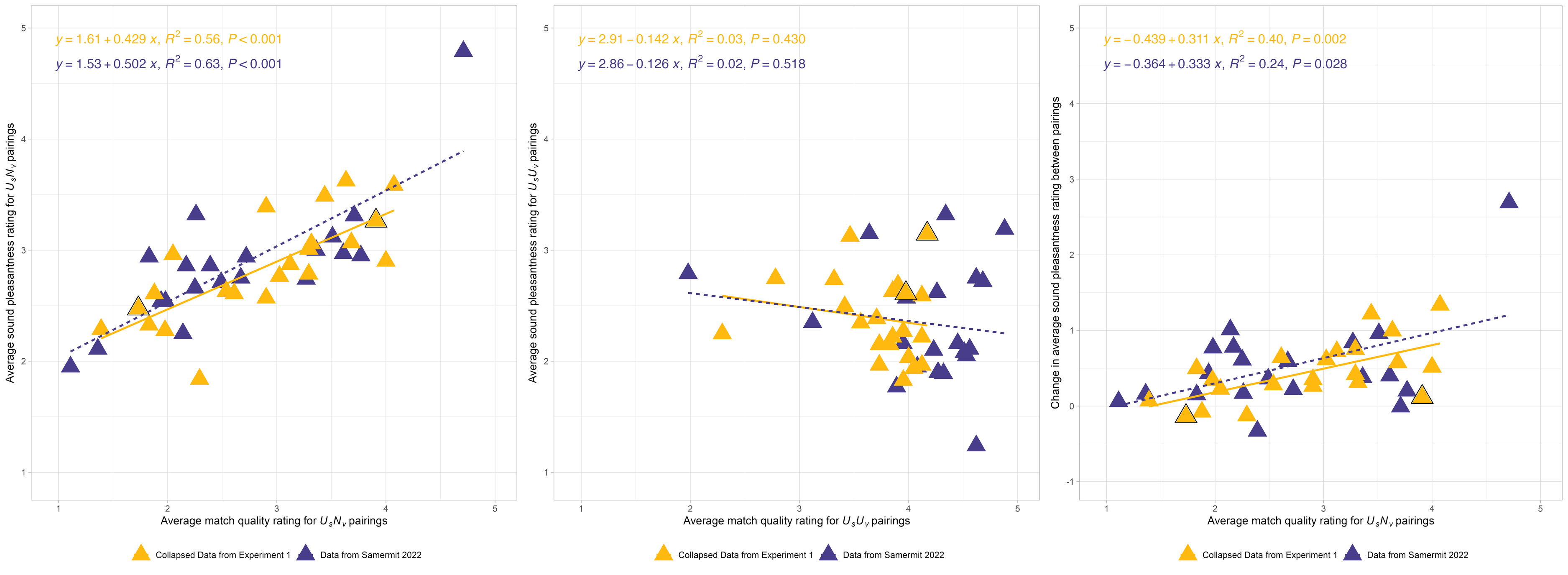

Supplement: S4 Fig — (A) The relationship between average sound pleasantness ratings for UsNv pairs versus average match quality ratings for UsNv pairs in Experiment 1 and Samermit et al., (2022). Data from Experiment 1 (across all participants) is indicated by yellow symbols and a solid line. Note, our pleasantness ratings were transformed from an 11-point scale to a 5-point scale to be congruent with Samermit et al., (2022). Data from Samermit et al., (2022) is indicated by purple symbols and a dashed line. Yellow symbols with a purple outline reflect movies that were borrowed from Samermit et al., (2022) to be used in Experiment 1. Each data point represents the mean rating across observers for one unpleasant sound, while the error bar reflects the standard error of the mean. (B) The relationship between average sound pleasantness ratings for UsUv pairs versus average match quality ratings for UsUv pairs in Experiment 1 and Samermit et al., (2022). (C) The relationship between the change in average sound pleasantness ratings across the two pairs (UsNv - UsUv) versus average match quality ratings for UsNv pairs in Experiment 1 and Samermit et al., (2022). The 22 data points represent the mean change for each of the unpleasant sounds and the error bars reflect the standard error of the mean across participants. (TIF) [file pone.0321594.s009.tif]

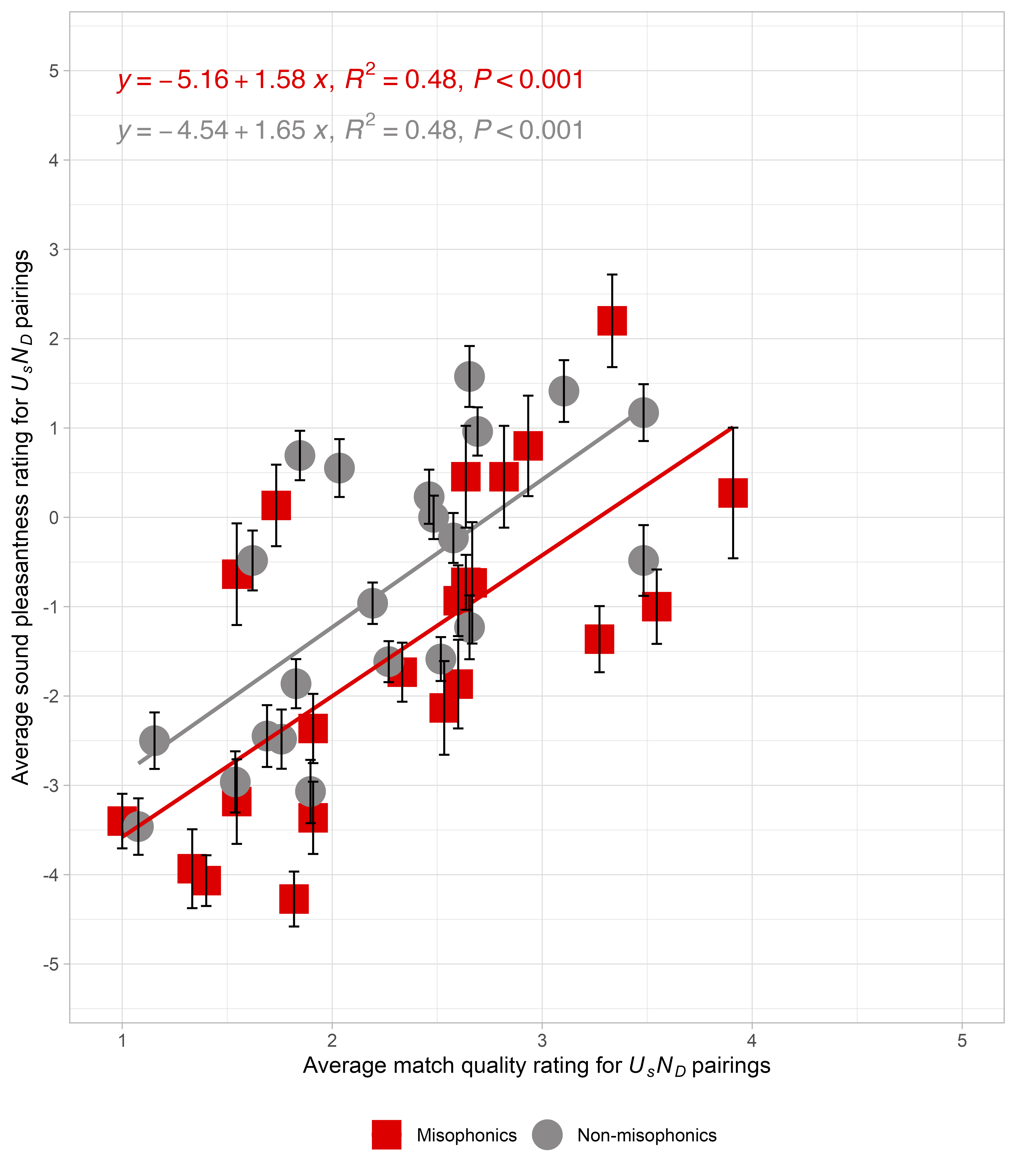

Supplement: S5 Fig — The relationship between average sound pleasantness ratings for UsND pairs and average match quality ratings for UsND pairs in Experiment 2. The averages are calculated across two mutually exclusive participant groups: Misophonics (red squares), and Non-misophonics (gray circles). The 22 data points represent individual unpleasant sounds. The error bars reflect the standard error of the mean. (TIF) [file pone.0321594.s010.tif]

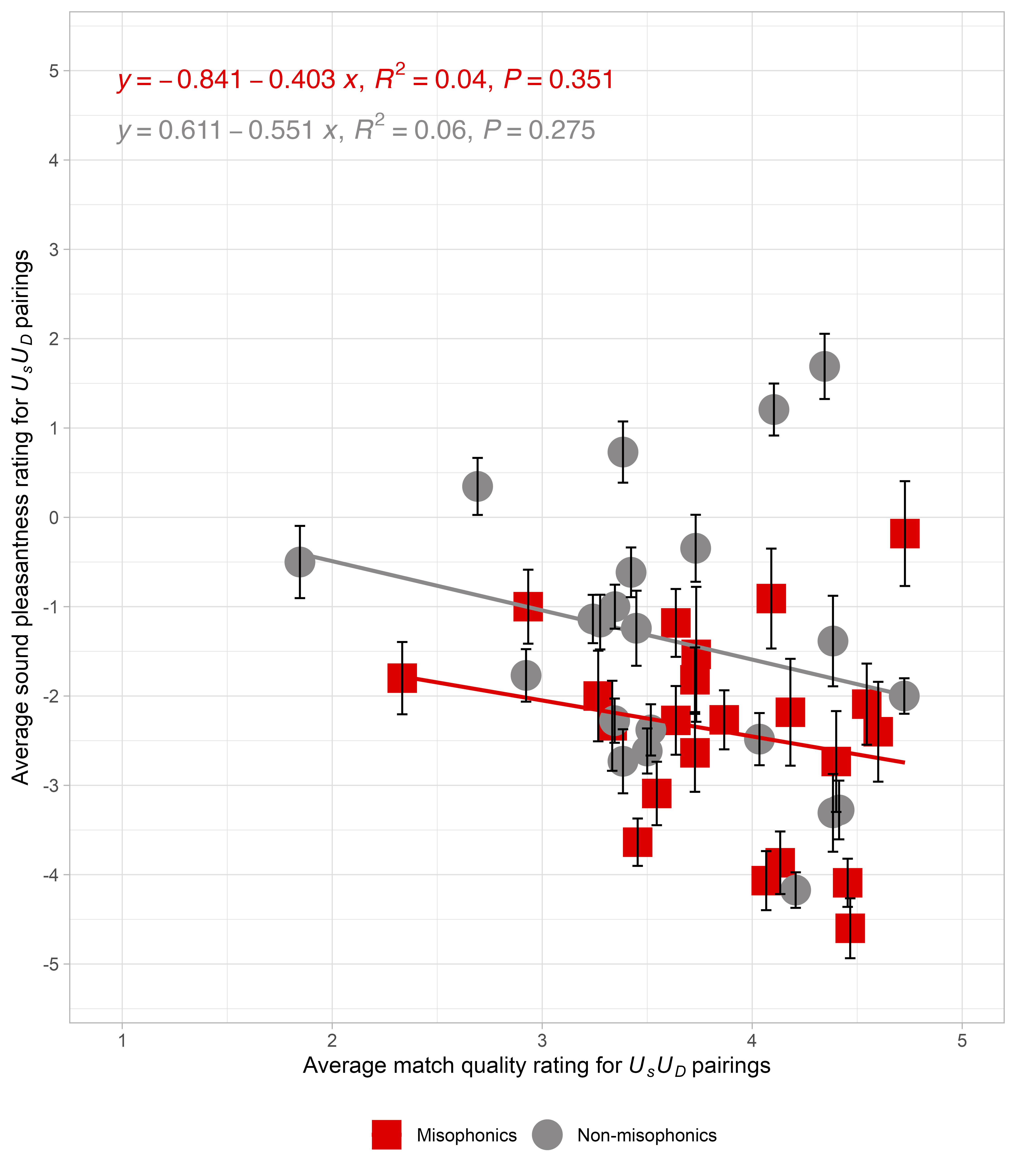

Supplement: S6 Fig — The relationship between average sound pleasantness ratings for UsUD pairs and average match quality ratings for UsUD pairs in Experiment 2. The averages are calculated across two mutually exclusive participant groups: Misophonics (red squares), and Non-misophonics (gray circles). The 22 data points represent individual unpleasant sounds. The error bars reflect the standard error of the mean. (TIF) [file pone.0321594.s011.tif]

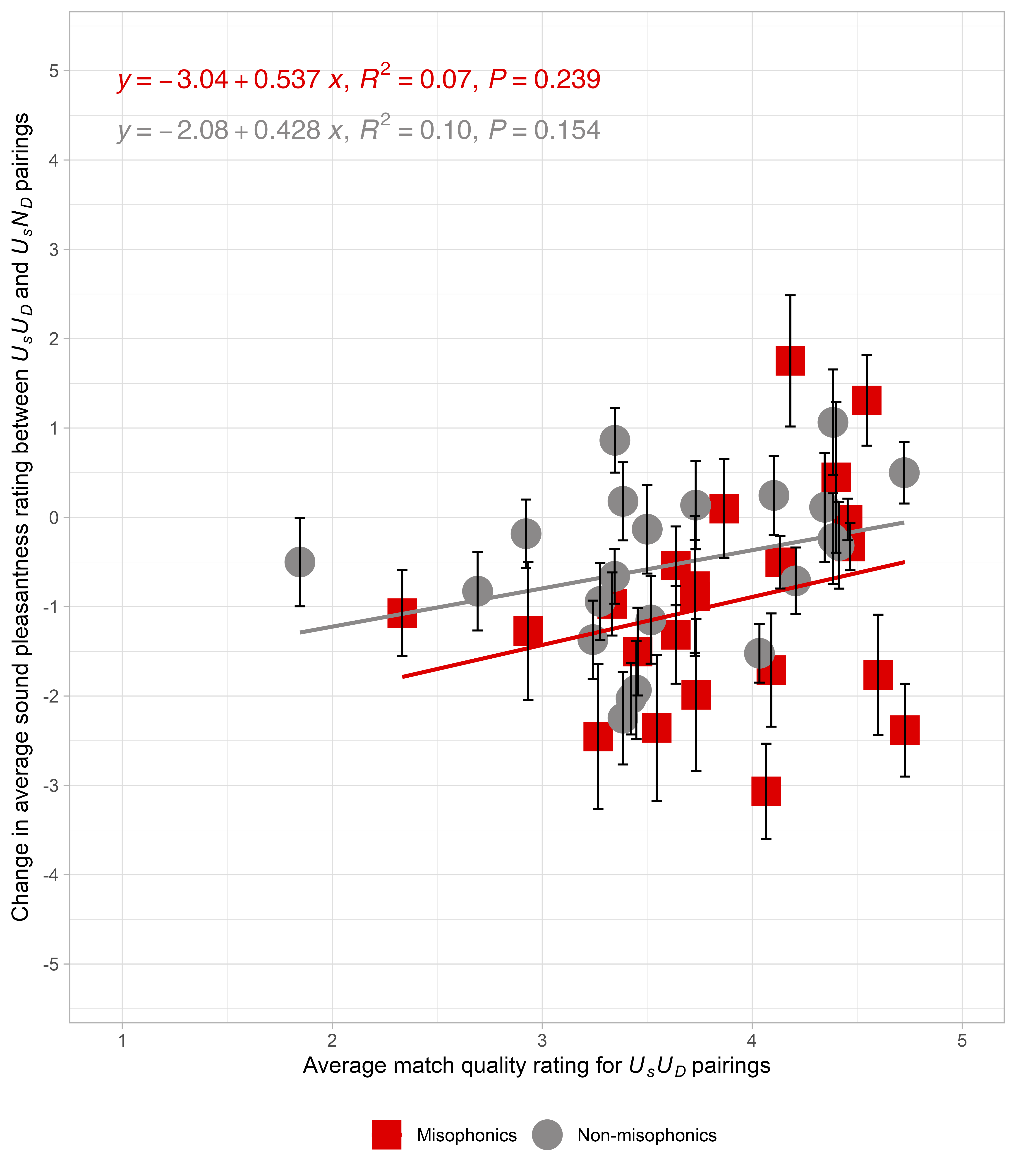

Supplement: S7 Fig — The relationship between the change in average sound pleasantness ratings between the two description conditions, and the average match quality ratings for UsUD pairs in Experiment 2. The averages are calculated across two mutually exclusive participant groups: Misophonics (red squares), and Non-misophonics (gray circles). The changes are calculated by subtracting the average pleasantness rating the sound receives in UsND pairing from the rating the sound receives in UsUD pairing. The 22 data points represent individual unpleasant sounds. The error bars reflect the standard error of the mean. (TIF) [file pone.0321594.s012.tif]

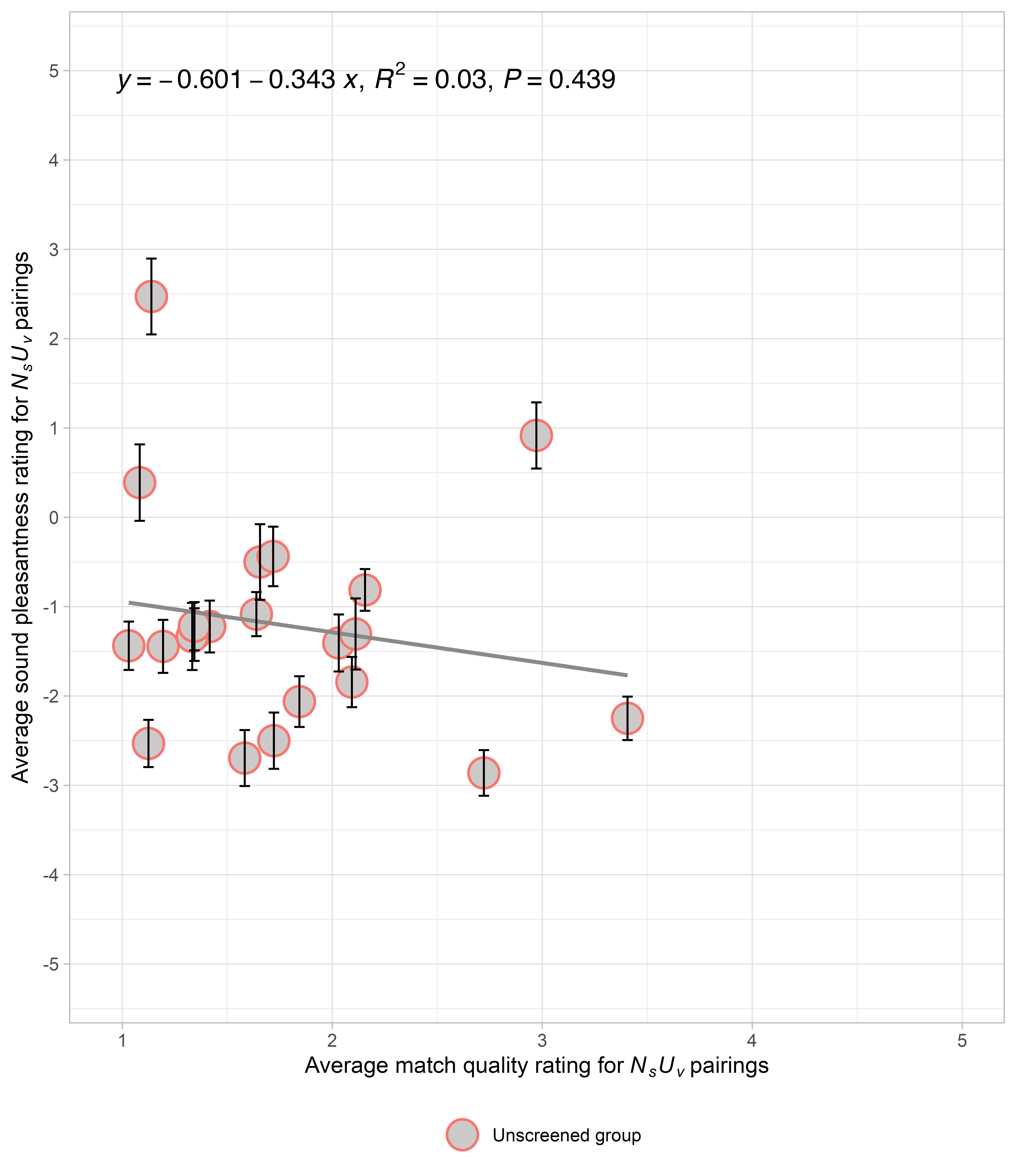

Supplement: S8 Fig — The relationship between average sound pleasantness ratings for NsUv pairs and average match quality ratings for NsUv pairs in Experiment 3A. The averages are calculated across all of the listeners in this unscreened group, irrespective of misophonic status. The 22 data points represent individual neutral sounds. The error bars reflect the standard error of the mean. (TIF) [file pone.0321594.s013.tif]

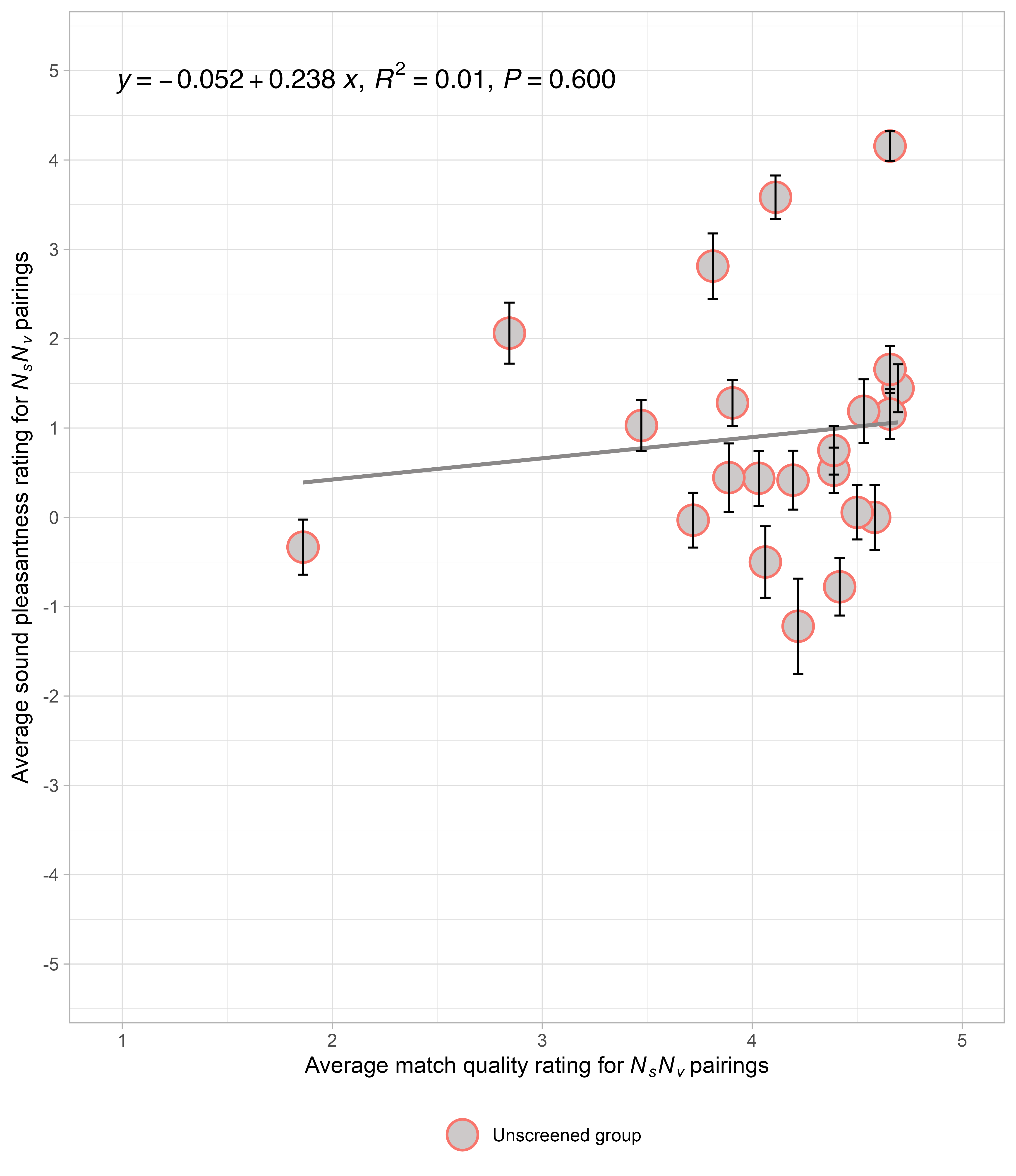

Supplement: S9 Fig — The relationship between average sound pleasantness ratings for NsNv pairs and average match quality ratings for NsNv pairs in Experiment 3A. The averages are calculated across all of the listeners in this unscreened group, irrespective of clinically significant misophonia status. The 22 data points represent individual neutral sounds. The error bars reflect the standard error of the mean. (TIF) [file pone.0321594.s014.tif]

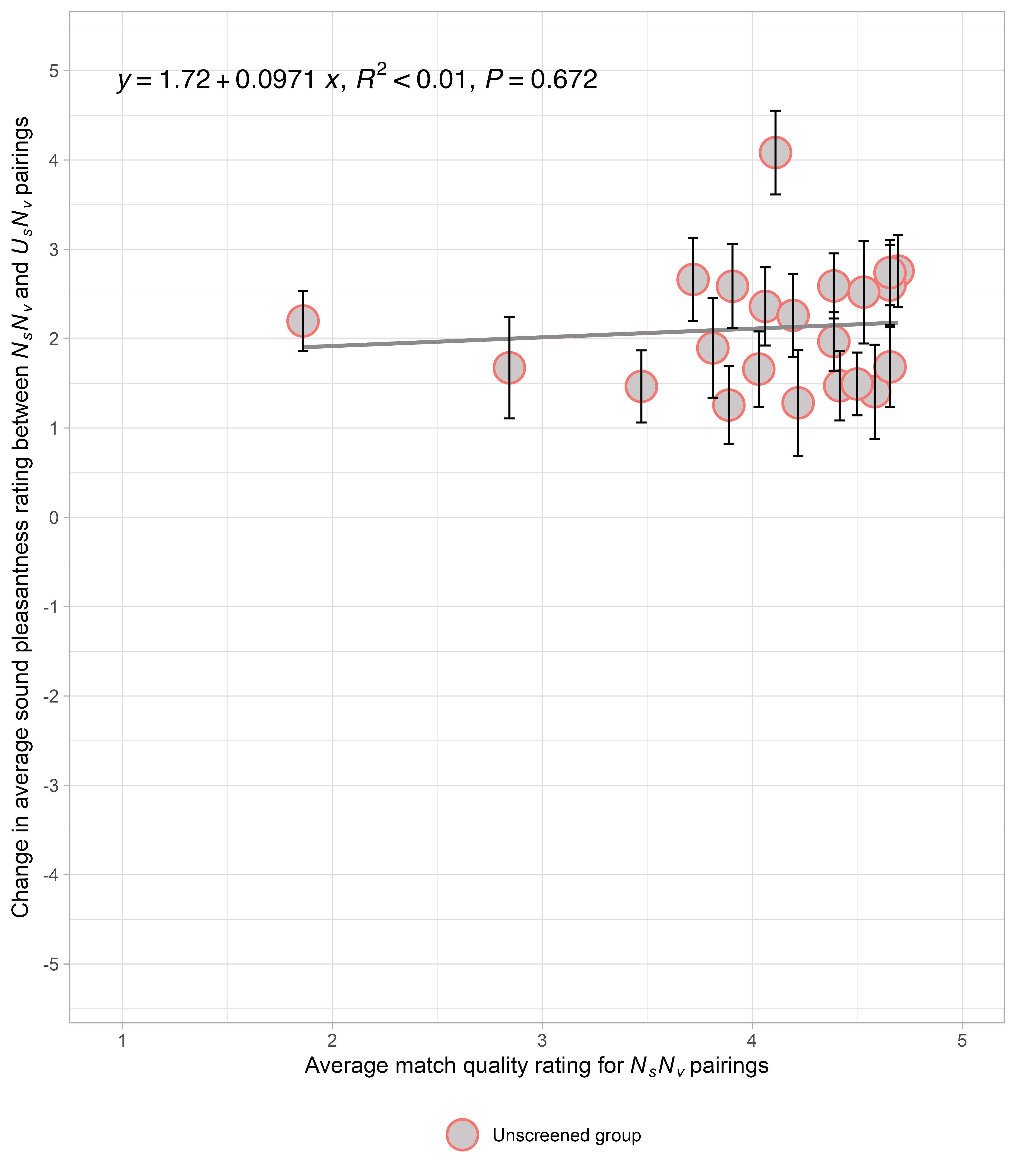

Supplement: S10 Fig — The relationship between the change in average sound pleasantness ratings between the two audio-video conditions, and the average match quality ratings for NsNv pairs in Experiment 3A. The averages are calculated across all listeners in this unscreened group, irrespective of misophonia status. The changes are calculated by subtracting the average pleasantness rating the sound receives in NsUv pairing from the rating the sound receives in NsNv pairing. The 22 data points represent individual neutral sounds. The error bars reflect the standard error of the mean. (TIF) [file pone.0321594.s015.tif]
